# Economic Evaluation of Inguinal Versus Ilio-inguinal Lymphadenectomy for Patients with Stage III Metastatic Melanoma to Groin Lymph Nodes: Evidence from the EAGLE FM Randomized Trial

**DOI:** 10.1245/s10434-025-17040-2

**Published:** 2025-02-27

**Authors:** Rashidul Alam Mahumud, Chi Kin Law, Daniel Ariza Ospino, Johannes H. W. de Wilt, Barbara L. van Leeuwen, Chris Allan, Vinicius de Lima Vazquez, Rowan Pritchard Jones, Julie Howle, Barbara Peric, Andrew J. Spillane, Rachael Lisa Morton

**Affiliations:** 1 NHMRC Clinical Trials Centre, Faculty of Medicine and Health, The University of Sydney, Camperdown, NSW Australia; 2 Melanoma and Skin Cancer Trials Limited, Melbourne, VIC Australia; 3 Department of Surgery, Radboud University Medical Center, Nijmegen, The Netherlands; 4 Division of Surgical Oncology, Department of Surgery, University Medical Center Groningen, University of Groningen, Groningen, The Netherlands; 5 Faculty of Medicine, Mater Clinical School, University of Queensland, Brisbane, QLD Australia; 6 Molecular Oncology Research Center, Barretos Cancer Hospital, Barretos, São Paulo, Brazil; 7 Department of Surgery of Melanoma and Sarcoma, Barretos Cancer Hospital, São Paulo, Brazil; 8 Department of Molecular and Clinical Cancer Medicine, Institute of Systems, Molecular and Integrative Biology, University of Liverpool, Liverpool, UK; 9 Mersey and West Lancashire Teaching Hospitals NHS Trust, Prescot, Knowsley, UK; 10 Sydney Medical School, The University of Sydney, Camperdown, NSW Australia; 11 Institute of Oncology Ljubljana, Zaloska 2, 1000 Ljubljana, Slovenia; 12 Faculty of Medicine, University of Ljubljana, Vrazov trg 2, 1000 Ljubljana, Slovenia; 13 Translational Research Hub, Poche Centre, Melanoma Institute Australia, The University of Sydney, Wollstonecraft, NSW Australia; 14 Breast and Melanoma Surgery Unit, Royal North Shore Hospital, St Leonards, NSW Australia; 15 Mater Hospital, Wollstonecraft, NSW Australia

**Keywords:** Cost-utility analysis, Quality-adjusted life years, Randomized controlled trials, Melanoma, Inguinal lymphadenectomy, Ilio-inguinal lymphadenectomy

## Abstract

**Purpose:**

We compared health outcomes and costs of inguinal lymphadenectomy (IL) versus ilio-inguinal lymphadenectomy (I-IL) for removal of metastatic melanoma to lymph nodes of the groin in adults with stage III melanoma.

**Methods:**

A within-trial cost-utility analysis was performed alongside an international randomized trial (EAGLE-FM) with 36 months follow-up from a health system perspective. Healthcare costs were measured by using trial records, and effectiveness measured in quality-adjusted life years (QALYs). Deterministic sensitivity analyses assessed the impact of changes in costs or quality of life on overall results. Statistical bootstrapping was employed to estimate confidence intervals around the cost-utility ratio.

**Results:**

Among 98 trial participants (IL *n *= 50, I-IL *n* = 48), with no pelvic or distant disease clinically or on PET/CT imaging, the mean life years saved for those randomized to IL showed a small but nonsignificant increase of 0.05 years compared with those in the I-IL group (2.56 vs. 2.51 years, 95% confidence interval [CI] –0.78 to 0.87). The mean difference in QALYs gained showed a small but nonsignificant increase of 0.04 QALYs (1.95 vs. 1.91, 95% CI –0.49 to 0.57). The mean hospital stay among IL patients was 6.16 days, 1.24 days shorter than I-IL patients (7.40 days) at 36 months follow-up. Mean per-patient healthcare costs of IL surgery were AU$6938 lower than for I-IL surgery ($26,555 vs. $33,493, 95% CI –$24,360 to $10,484). Inguinal lymphadenectomy was slightly more effective and slightly less expensive) over I-IL; a finding supported by 81% of bootstrapped estimates and upheld across sensitivity analyses.

**Conclusions:**

Our study indicates that less extensive IL surgery might be the preferred surgical strategy for metastatic melanoma to the groin when PET/CT imaging shows no pelvic disease. This surgery is likely to improve quality-adjusted survival and may reduce healthcare costs; however, the differences noted in EAGLE-FM were limited by a small sample size and were not statistically significant.

**Trial Registration:**

Clinicaltrials.gov NCT02166788; anzctr.org.au ACTRN12614000721606.

**Supplementary Information:**

The online version contains supplementary material available at 10.1245/s10434-025-17040-2.

The spread of metastatic melanoma to the groin lymph nodes is common in patients with stage III melanoma.^1^ Whilst groin lymphadenectomy has been standard first-line treatment for many years, whether to do an inguinal lymphadenectomy (IL) or ilio-inguinal lymphadenectomy (I-IL) for patients when the metastatic melanoma appears clinically and radiologically confined to the inguinal area, is controversial with strong views held by surgeons from different institutions.^2^ The benefits of more extensive and more complicated I-IL include the removal of occult pelvic disease to prevent further recurrence and more accurate staging.

The Evaluation of Groin Lymphadenectomy for Metastatic Melanoma (EAGLE FM) randomized trial was designed in 2014 to determine the clinical and cost-effectiveness of IL versus I-IL. Since then, there have been rapid advances in systemic therapy, including targeted, adjuvant immunotherapy and neoadjuvant therapy with significant improvements in progression-free survival.^3–12^ The results of the MSLT2^13^ and DeCOG SLT^14^ trials ended the role for completion lymphadenectomy after sentinel node biopsy. EAGLE FM was subsequently closed owing to futility, with only 101 of a target 634 patients recruited; however, the trial Data Safety Monitoring Committee recommended the follow-up of randomized participants be continued to obtain the only known comparative estimates of effectiveness.

There is a trend toward deescalating surgery; in at least one study, removal of the index lymph node rather than full IL showed early compelling results for patients with major pathological response to neoadjuvant immune therapy.^7^ Nevertheless, in many parts of the world, the lack of systemic therapies makes the impact of surgery extent vital for patient outcomes, including disease-free survival, quality of life (QoL), and for health system costs. Despite surgery being the least rigorously evaluated cancer treatment modality, evidence of cost-effectiveness in medical and surgical procedures, and impact on QoL and survival remain critical yet underexplored.^15,16^

In Australia, there currently exists a moderate cost differential of $723 for the operative procedure to add an ilio-inguinal lymphadenectomy (I-IL) to the inguinal lymphadectomy (IL) (Medicare Benefits Schedule item number 30330 for IL plus 36502 for I-IL). However, comprehensive healthcare costs and patient outcomes following both surgical procedures have not been thoroughly examined. Decreased QoL due to postoperative morbidity and recurrence, along with the potential QoL decline with more extensive surgery (I-IL), are significant economic endpoints warranting investigation. This economic evaluation provides evidence to inform clinical practice guidelines and policy, aiding clinical decision-making in settings where lymphadenectomy is indicated, and systemic therapies are not yet available. Adopting an Australian health system perspective, this study aimed to compare the within-trial cost-effectiveness of IL versus I-IL surgical approaches for the removal of metastatic melanoma in the lymph nodes of the groin.

## Methods

### Study Population

Participants were enrolled and randomized in the Evaluation of Groin Lymphadenectomy Extent For metastatic Melanoma (EAGLE-FM) trial.^17^ Trial participants were recruited based on the following inclusion criteria: aged ≥ 15 years; primary cutaneous melanoma or stage III melanoma with no known primary tumor and metastatic melanoma in one or multiple inguinal nodes; no pelvic nodal or distant disease clinically or on PET/CT imaging; patient treatment history, including adjuvant systemic therapy; able to provide written, informed consent; and willing to return to the centre for follow-up examinations and procedures.^17,18^ All patients were randomized and underwent lymphadenectomy surgery no more than 90 days after diagnosis of inguinal lymph node involvement.

### Setting and Location

The EAGLE FM trial commenced at 17 tertiary melanoma treatment centers and surgical oncology clinics in six countries: Australia (7 sites), Slovenia (1-site), UK (4 sites), Italy (1-site), Brazil (2 sites), and the Netherlands (2 sites), coordinated by Melanoma and Skin Cancer Trials Limited (MASC Trials: https://www.masc.org.au/) [formerly Australia and New Zealand Melanoma Trials Group (ANZMTG)].

### Surgical Treatment Intervention and Control

The IL (intervention) group of patients underwent a surgical procedure to remove easily accessible superficial lymph nodes in the groin. I-IL (control) group approach consisted of the removal of the same superficial lymph nodes during an IL, with the addition of surgical excision of ipsilateral pelvic lymph nodes. Surgeons performing the IL typically remove around 11 superficial lymph nodes from the groin over a span of 1 to 2 h. In contrast, the I-IL group results in the removal of approximately 22 lymph nodes up to 3 h.

Clinical Practice Guidelines advocated a minimum of IL for proven melanoma in groin lymph nodes.^19^ They also recommend considering I-IL with radiological evidence of pelvic lymph node metastases on imaging, gross inguinal clinical involvement, ≥ 3 inguinal nodes involved, or the presence of clinically suspicious lymph nodes high in the groin. Both surgical approaches were therefore considered standard of care for metastatic groin nodes at the time of trial design. Irrespective of the above indicators for considering I-IL, in this trial patients with isolated disease in the superficial inguinal area on clinical examination and PET/CT scan were randomized to IL or I-IL. Patients were randomized 1:1, at their initial baseline visit using a 24/7 web-based randomization system implemented on the MASC Trials website (https://www.masc.org.au/).

### Study Perspective

Adopting an Australian health system perspective, a preplanned within-trial cost-utility analysis was conducted alongside this prospective, international, multi-center, phase III, two-armed, noninferiority, randomized clinical trial. The trial was registered with ClinicalTrials.gov (Identifier: NCT02166788), and with anzctr.org.au (Trial Id: ACTRN12614000721606); the full trial design is described elsewhere.^17^ Data on included participants were analyzed by adopting an intention-to-treat basis. This economic evaluation was based on the Consolidated Health Economic Evaluation Reporting Standards (CHEERS) 2022 recommendations (Supplementary Appendix Table S1.1).^20^

### Outcome Selection, Measurement, and Valuation

Overall survival, QoL, and quality-adjusted survival were chosen as the relevant trial outcomes. We assessed utility-based QoL by using scores from the EQ-5D-5L instrument. The EQ-5D-5L questionnaire was developed by the EuroQol Group and has been a widely used generic health-related quality-of-life instrument with five dimensions (mobility, self-care, usual activities, pain/discomfort, and anxiety/depression) and five self-rated severity response levels (e.g. no, slight, moderate, severe, unable to/extreme problems) for each dimension.^21^ We used Australian EQ-5D-5L weights to measure utility values at these time points.^22^ We adopted the population-based approach to estimate quality-adjusted survival (QAS), which involved estimating survival probabilities and quality of life (the preferred health utility indices) at the beginning and end of each measurement period.^23^ Survival probabilities were estimated using the Kaplan-Meier estimate.^24^

The Kaplan-Meier estimator of the survival function was defined as

1 $$\widehat{{S(t)}} = \left\{ {\begin{array}{*{20}l} 1 \hfill & {t < t_{1} } \hfill \\ {\prod\limits_{{t_{i} \le t}} {\left[ {1 - \frac{{d_{i} }}{{N_{i} }}} \right]} } \hfill & {t_{1} \le t,} \hfill \\ \end{array} } \right.$$

where ${d}_{i}$ equal to the number of events at time point *i*, ${N}_{i}$ was the number of patients at risk at time point *i and* ${t}_{i}$ was the time point with an event or censoring.

Life years (LYs) were calculated based on the survival probabilities using Kaplan-Meier estimator and trial follow-up times after surgery using a restricted mean survival approach, which was represented by the area under the survival curve^25^:

$$Undiscounted LYs =$$

2 $$\widehat{{\mu }_{T}}= {\int }_{0}^{T}\widehat{S\left(t\right)} dt=\sum_{{t}_{i }\le T=0}^{{T}_{k}} {S}_{w}^{*}\times \left({t}_{i+1}-{t}_{i}\right)$$

Where T total trial follow-up time, *t*_*i*_ specific trial follow-up time [*i* = baseline (0), 3 months (0.25 yr), 6 months (0.5 yr), 9 months (0.75 yr), 12 months (1 yr), 15 months (1.25 yr), 18 months (1.5 yr), 21 months (1.75 yr), 24 months (2 yr), 30 months (2.5 yr), 36 months (3 yr)], and ${S}_{w}^{*}$ equal to the survival probability between ${t}_{i+1}$ and ${t}_{i}$ time points.

The QAS was calculated by using the population-based approach at different time points in the clinical trial^26^:

3 $$Undiscounted\,\,QAS= \sum_{i=0}^{k}\left[\frac{\left({Q}_{i}+{Q}_{i+1}\right)}{2} \times \frac{\left({S}_{i+1}+{S}_{i}\right)}{2}\right] \left({t}_{i+1}-{t}_{i}\right)$$

where ${Q}_{i}$ are the mean utility score (for details, Supplementary Appendix file S2). Intuitively, this is the quality of life averaged over two periods’ times the average survival probability times the length of the time period of interest. The total QAS for each surgical group’s trial duration are a summation of QAS calculations for each follow-up time point (Eq. 3, details Supplementary appendix Table S1.2). Hence, the quality-adjusted survival curve is formed by plotting against time (t) the product of the mean quality-of-life score of patients alive at time t and the probability of surviving time t. The unit of QAS is quality-adjusted life year (QALY), where the utility scores were used for health-related quality of life measure and the time unit is 1 year.^27^ A 5% discount rate for outcomes was applied after one year according to Australian guidelines.^28^

### Measurement and Valuation of Healthcare Use and Costs

Direct medical costs for the IL and I-IL procedures and medicines were determined based on trial record. Healthcare use was valued by using Medicare claims data (Medical Benefits Schedule and Pharmaceutical Benefits Scheme item numbers, Supplementary Appendix Table S1.3). All related medical costs for clinical care in the first 36 months were captured, including primary care practitioner, specialist doctor (e.g., surgical oncologist), palliative care professional (i.e., doctor or nurse), allied health (e.g., dietician or social worker) visits; diagnostic tests or scans; systemic therapies and radiation therapy; and hospitalizations (length of stay). Hospitalization was valued according to the Australian National Hospital Cost Data Collection report.^29^ A 5% discount rate per year was applied after 1 year.^28^ Healthcare use data were collected from all randomized participants, including those from countries outside of Australia.

### Cost-utility Analysis

The health outcomes for patients with melanoma were measured using LYs/QALYs gained over 36 months, calculated at the patient level from survival data and EQ-5D-5L health state scores. An incremental cost-effectiveness/cost-utility ratio (ICER/ICUR) was calculated as the total cost for the intervention (IL: less extensive surgery) minus the total cost for the control (I-IL: more extensive surgery) divided by the total LYs/QALYs of the intervention minus the LYs/QALYs of the control:

4 $$\text{ICER}/\text{ICUR}= \frac{{\text{Intervention }(\text{IL surgery})}_{\text{costs}}- {\text{Control }(\text{I}-\text{IL surgery})}_{\text{costs}}}{{\text{Intervention }(\text{IL surgery})}_{\text{QALYs}/\text{LYs}}-{\text{Control }(\text{I}-\text{IL surgery})}_{\text{QALYs}/\text{LYs}}}$$

We used a willingness-to-pay threshold value (AU$ 50,000) for the ICUR per QALY gained (and ICER per LY gained), a generally accepted level for “value for money” in health technology assessments in Australia. In the context of the cost-effectiveness decision, a program or intervention is economically viable (i.e., acceptable value for money) when the incremental cost-effectiveness/cost-utility ratio per QALY gained is below the willingness-to-pay threshold value. In addition, we calculated the incremental net monetary benefit, as the incremental QALYs multiplied by the willingness-to-pay threshold value, minus the incremental cost of IL surgery compared with I-IL.

### Statistical Analysis

Costs and effects were calculated using within-trial patient-level data. We estimated the mean life-years for both surgical groups to understand the long-term survival for patients at 36 months. For any missing data, we applied a single imputation method with a single predicted value for estimating healthcare use and health utilities, such as the mean, by trial allocation group for a given case.^30^ Data are presented as means with standard deviations and mean differences between the two surgery groups with 95% CIs. The QALYs are expressed as a point estimate (mean value) estimated using Kaplan-Meier survival analysis. In addition, bootstrapping with 1000 replications was used to assess the statistical significance of differences in healthcare use, costs and LYs and QALYs with 95% CIs in. All analyses and data management were performed by using Stata SE/15 software.

### Sensitivity Analyses

We assessed the robustness of estimates using a series of deterministic (one-way) sensitivity analyses (Supplementary Appendix Table S1.4). Analyses considered the base case with a 10–30% increase or decrease in the major direct medical costs. For example, we increased and decreased medical costs for health services, including general practitioners, specialist doctors, diagnostic tests, and hospitalizations. In addition, other sensitivity analyses were performed reducing the discount rate for costs and benefits to 3 or 3.5% and applying different utility values for the EQ-5D-5L using the 2022 Australian tariffs.^31^

## Results

### Participant Characteristics

Ninety-eight of 101 trial participants (50 randomized to IL and 48 to I-IL surgery) were included in the economic evaluation. Of the three participants excluded from the economic evaluation, two withdrew, and one did not complete EQ-5D-5L assessments. The patient cohort consisted of 35 patients from Australia, 24 from Slovenia, 17 from Brazil, 9 from UK, 9 from the Netherlands, and 4 from Italy (Table 1). At baseline, Breslow thickness was documented for the majority of patients in both surgery groups, with values available for 86% of patients in IL surgery (43 patients) compared with 81% (39 patients) in I-IL surgery. Among patients with recorded Breslow thickness, intermediate melanomas (> 1.00–4.00 mm) were the most prevalent, observed in 65% of IL surgery patients compared with 56% of I-IL surgery patients. Thick melanomas (> 4.00 mm) were more frequently observed in the I-IL surgery (28%) compared with the IL surgery (21%) (Table 1).

**Table 1:** Baseline characteristics of trial participants

Characteristics	Inguinal lymphadenectomy (*n* = 50)	Ilio-inguinal lymphadenectomy (*n *= 48)	*p*
Gender, n (%)			
Males	23 (45.20)	21 (43.75)	0.840
Females	27 (54.80)	27 (56.25)	
Age (yr), mean (SD)	56.3 (11.79)	56.2 (13.37)	0.969
Age group, n (%), yr			
27 to 40	5 (10.00)	5 (10.42)	0.897
41 to 50	13 (26.00)	13 (27.08)	
51 to 60	14 (28.00)	10 (20.83)	
61 to 70	13 (26.00)	12 (25.00)	
71 to 83	5 (10.00)	8 (16.67)	
Body mass index (BMI), mean (SD)	26.91 (4.47)	26.22 (5.23)	0.487
BMI category			
Underweight (< 18.5)	–	1 (2.08)	0.395
Healthy weight (18.50–24.99)	16 (32.00)	20 (39.58)	
Overweight (25.00–29.99)	22 (44.00)	15 (31.25)	
Obese (≥ 30.00)	11 (22.00)	13 (27.08)	
Missing observation	1 (2.00)		
Study sites			
The Netherlands	6 (12.00)	3 (6.25)	0.827
Slovenia	12 (24.00)	12 (25)	
Italy	3 (6.00)	1 (2.08)	
United Kingdom	4 (8.00)	5 (10.42)	
Brazil	8 (16.00)	9 (18.75)	
Australia	17 (34.00)	18 (37.5)	
EQ-5D-5L utility index, mean (SD)	0.79 (0.20)	0.78 (0.21)	0.809
Breslow Thickness (millimetres, mm)			
Thin (≤ 1.00 mm)	6 (13.95)	6 (15.38)	0.695
Intermediate (> 1.00 to 4.00 mm)	28 (65.12)	22 (56.41)	
Thick (> 4.00 mm)	9 (20.93)	11 (28.21)	

The probability value (*p*-value) was generated using two samples independent t-test or chi- squared (or Fisher’s exact) test where appropriate

*SD* Standard deviation

### Health Outcomes

**Fig. 1:**
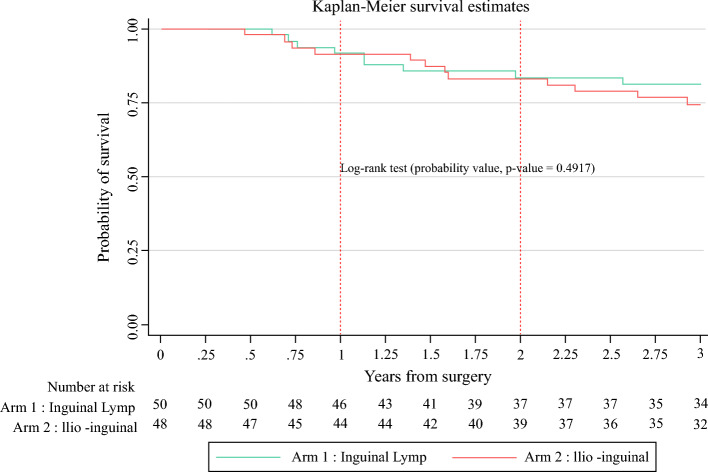
Overall survival

At the end of 36 months, the probability of survival among patients receiving IL surgery was slightly but not statistically significantly higher than those receiving I-IL surgery (Fig. 1) (log-rank test, probability value = 0.4947). Patients with IL surgery accrued 0.05 (95% CI −0.78 to 0.87) more life years (LYs) than patients in the I-IL surgery (2.56 vs. 2.51 years).

Eighty-nine participants completed the EQ-5D-5L questionnaire (quality of life) at baseline (IL = 47 patients [94%] and I-IL = 42 patients [88%]). Quality of life data were subsequently collected for 79 participants at the 12-month timepoint (IL = 40 patients [91%] and I-IL = 39 patients [89%]), 68 participants at the 24-month (IL = 33 patients [85%] and I-IL = 35 patients [88%]), and 60 participants at the 36-month (IL = 30 patients [79%] and I-IL = 30 patients [83%]) follow-up (Supplementary Appendix Fig. S1). The average EQ-5D utility scores among patients in the IL and I-IL groups were 0.79 and 0.78 at baseline, respectively (Table 1). At 36-month follow-up, the average utility score in the IL surgery group (0.91) was slightly higher than the I-IL surgery (0.88) but not statistically significantly higher (0.03; 95% CI −0.06 to 0.12). It is worth noting that this difference is close to the range of minimal clinically important difference (0.037 to 0.069), which is typically considered significant to a patient.^32^

**Fig. 2:**
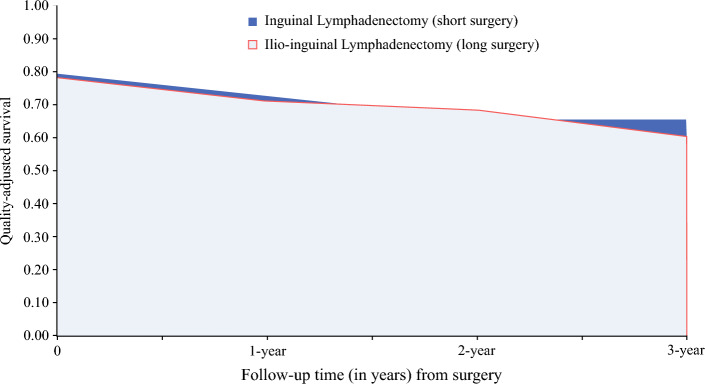
Quality-adjusted survival more than 3 years postsurgery

The area under QAS curve represents the total QALYs per trial allocation group (Fig. 2). A higher area under the QAS curve for the IL surgery group suggests that patients in this group lived longer and experienced a better quality of life during their survival period compared with I-IL surgery group (Fig. 2). Patients in the IL surgery group had 0.04 higher mean QALYs than the I-IL surgery group (1.95 vs. 1.91) after 36 months (95% CI −0.49 to 0.57; Table 2).

**Table 2:** Cost-utility analysis from a health system perspective

Cost-utility/effectiveness analysis at 3 years	Inguinal	Ilio-inguinal lymphadenectomy (*n *= 48)
	lymphadenectomy	
	(*n *= 50)	
Total direct medical cost per patient, mean (SD) (AU$)	$ 26,555 (42,498)	$ 33,493 (45,885)
Life years (LYs) per patient, mean (SD)	2.56 (1.72)	2.51 (1.74)
Quality-adjusted life years (QALYs) per patient, mean	1.95	1.91
Incremental cost per patient, mean (95% CI) (AU$)	−$ 6938 ($ − 24,360 to $ 10,484)^b^	
Incremental life years gained, mean (95% CI)	0.05 (− 0.78 to 0.87)	
Incremental benefit per patient: QALY gained, mean (95% CI)	0.04 (− 0.49 to 0.57)	
Cost-effectiveness^a^ for LY gained	Inguinal lymphadenectomy is dominant	
Cost-effectiveness^a^ for QALY gained	Inguinal lymphadenectomy is dominant	
Incremental net benefit per LY gained	$ 9438	
Incremental net benefit per QALY gained	$ 8938	

A generalized linear model (GLM) fitted to the data with a gamma family distribution

*SD* Standard deviation

^a^At a willingness-to-pay threshold of AU$50,000 per QALY/LY gained

^b^Bootstrap 95% confidence intervals (CIs) were computed using 1000 replications with

### Healthcare Utilization

Table 3 shows the distribution of healthcare use per patient by health service type and trial allocation. Over the 36-month follow-up period, healthcare use in most categories was lower for patients in the IL than the I-IL surgery group. Specifically, the average hospital length of stay was slightly shorter (1.24 days) among patients in the IL surgery than in the I-IL (6.16 vs. 7.40 days). Among total healthcare use, approximately one-third was for diagnostic tests or scans in both groups (Table 3). However, patients undergoing I-IL surgery required 8% more specialist doctor care (e.g., medical or radiation oncologists) than patients in the IL surgery (23 vs. 15%). The average general practitioner consultations were almost double among patients in the IL surgery than in the I-IL surgery (12 vs. 7%).

**Table 3:** Mean volumes of healthcare use per patient by trial allocation at 3 years follow-up

Category	Items	Inguinal lymphadenectomy (*n* = 50)	Ilio-inguinal lymphadenectomy (*n *= 48)	Differences in healthcare use
		Healthcare use, mean (SD)	Healthcare use, mean (SD)	Mean (95% CI)^§^
General practitioner	General practitioner clinic visit	4.57 (8.37)	3.61 (5.55)	0.97 (− 1.82 to 3.75)
	General practitioner home visit	0.08 (0.34)	0.08 (0.40)	− 0.003 (− 0.15 to 0.15)
	General practitioner (telephone)	0.30 (0.79)	0.09 (0.36)	0.21 (− 0.02 to 0.44)
	Other health professional visits	2.88 (7.22)	1.15 (3.23)	1.73 (− 0.4 to 3.86)
	Subtotal consultations	7.83 (11.48)	4.92 (6.57)	2.91 (− 0.74 to 6.55)
Specialist doctor	Radiation oncologist	0.80 (2.04)	1.06 (2.50)	− 0.26 (− 1.2 to 0.67)
	Medical oncologist	5.80 (9.37)	9.32 (12.52)	− 3.52 (− 7.95 to 0.92)
	Surgical oncologist	0.38 (1.19)	0.54 (1.68)	− 0.16 (− 0.74 to 0.42)
	Other cancer specialist visits	2.97 (4.12)	5.44 (8.39)	− 2.47 (− 5.2 to 0.26)
	Sub-total specialist doctor consultations	9.95 (12.35)	16.36 (18.44)	− 6.41 (− 12.83 to 0.01)
Palliative care	Palliative care professional (i.e., doctor, nurse)	0.02 (0.14)	0.04 (0.20)	− 0.02 (− 0.09 to 0.05)
Allied health	Dietician	0.32 (1.32)	0.27 (0.82)	0.05 (− 0.37 to 0.47)
	Social worker	0.02 (0.14)	0.15 (0.87)	− 0.13 (− 0.38 to 0.13)
	Physiotherapist	3.80 (6.96)	2.46 (3.64)	1.34 (− 0.88 to 3.56)
	Psychologist	1.07 (3.15)	0.23 (0.69)	0.84 (− 0.1 to 1.78)
	Lymphoedema therapist	6.75 (10.65)	7.19 (15.2)	− 0.44 (− 5.64 to 4.77)
	Sub-total allied health consultations	11.96 (16.15)	10.29 (16.18)	1.67 (− 4.6 to 7.94)
Other surgery	Surgery (e.g. for local recurrence)	0.00 (0.00)	0.06 (0.32)	− 0.06 (− 0.14 to 0.02)
	Stereotactic radiosurgery (SRS)	0.36 (0.85)	1.17 (4.35)	− 0.8 (− 2.06 to 0.45)
	Sub-total other surgery	0.36 (0.85)	1.23 (4.35)	− 0.87 (− 2.12 to 0.39)
Diagnostic imaging and tests	Cytology testing (e.g., biopsy)	0.74 (1.24)	0.67 (1.12)	0.07 (− 0.4 to 0.55)
	MRI scan	1.78 (2.93)	1.4 (2.29)	0.38 (− 0.63 to 1.4)
	CT scan	2.42 (3.77)	3.02 (3.51)	− 0.6 (− 2.02 to 0.82)
	PET scan	1.80 (2.12)	2.4 (2.52)	− 0.6 (− 1.48 to 0.29)
	Blood test	8.62 (12.67)	9.48 (9.81)	− 0.86 (− 5.42 to 3.7)
	Ultrasound	1.20 (1.98)	0.98 (1.67)	0.22 (− 0.49 to 0.93)
	X-Ray	0.20 (0.73)	0.38 (1.36)	− 0.18 (− 0.6 to 0.25)
	Bone scan	0.04 (0.20)	0.042 (0.2)	0.002 (− 0.08 to 0.08)
	Mutation Testing	0.24 (0.48)	0.15 (0.36)	0.09 (− 0.06 to 0.25)
	Other diagnostic tests	0.30 (1.05)	1.27 (7.65)	− 0.97 (− 3.13 to 1.19)
	Sub-total diagnostic tests	17.34 (22.31)	19.77 (17.37)	− 2.43 (− 10.23 to 5.37)
Medicine	Medicine	2.20 (3.74)	3.79 (6.24)	− 1.59 (− 3.65 to 0.46)
Radiation therapy	Radiation therapy	2.20 (6.81)	2.31 (6.22)	− 0.11 (− 2.73 to 2.5)
Systemic therapies	Targeted therapy	5.28 (28.09)	4.92 (25.54)	0.36 (− 10.22 to 10.93)
	Chemotherapy	0.00 (0.00)	0.65 (2.29)	− 0.65 (− 1.26 to − 0.04)
	Immunotherapy	1.58 (4.43)	2.21 (7.62)	− 0.63 (− 3.08 to 1.82)
	Sub-total therapies	9.06 (30.01)	10.09 (27.34)	− 1.03 (− 12.4 to 10.34)
Hospitalization	Hospitalization Length of Stay (LOS) in days	6.16 (11.92)	7.40 (15.00)	− 1.24 (− 6.71 to 4.24)

*SD* Standard deviation; *GP* General practitioner; *PET* Positron emission tomography

^§^Bootstrap confidence intervals with 1000 replications

### Healthcare Costs

The average specialist doctor consultation costs per patient were significantly lower in the IL surgery group ($938) compared with I-IL surgery $1703 (–$765; 95% CI −1460 to −69), including per-patient radiation oncology costs ($697 vs. $1323; −$627; 95% CI −1233 to −20) (Table 4). Per-patient health service costs for IL surgery were on average $6938 lower (95% CI −$24,360 to $10,484) than for I-IL surgery ($26,555 vs. $33,493; Table 4). Hospitalization costs were the largest cost component for both surgery groups. Fifty-two percent of total treatment costs were incurred by hospitalizations among patients in the IL surgery group, compared with 49% in the I-IL surgery group. While the average per-patient hospitalization cost was lower for IL surgery than I-IL surgery ($13,922 vs. $16,434), the difference was not statistically significant (−$2512; 95% CI −$14,654 to $9630). The second-largest cost driver for both groups was systemic therapies and radiation therapy: $6617 per patient in the IL surgery and $7379 in the I-IL surgery. These costs accounted for 25% of total costs among patients in the IL surgery group and 22% among patients in the I-IL surgery group (Table 4). The average medicines cost per patient was $1980 lower in the IL surgery than in the I-IL surgery ($575 vs. $2553). This difference likely reflects the increased use of analgesics or patient-controlled analgesia, longer and deeper anaesthetics, and more intensive postoperative pain management in the I-IL surgery.

**Table 4:** Mean healthcare costs per patient by type of health service and trial allocation at 3 years follow-up

Categories	Items	Inguinal lymphadenectomy (*n *= 50)	Ilio-inguinal lymphadenectomy (*n *= 48)	Difference in costs
		Costs (AU$), mean (SD)	Costs (AU$), mean (SD)	Mean (95% CI)^*§*^
Index surgery	Index surgery^¥^	569.85	1103.55	–
General practitioner	General practitioner clinic visit	93.90 (172.09)	73.88 (114.65)	20.02 (− 37.31 to 77.35)
	General practitioner home visit	2.56 (11.01)	2.6 (12.63)	− 0.04 (− 4.84 to 4.76)
	General practitioner (telephone)	2.88 (7.60)	0.87 (3.56)	2.01 (− 0.24 to 4.25)
	Other health professionals visit	233.24 (533.90)	141.82 (371.53)	91.42 (− 86.12 to 268.96)
	Sub-total consultations	332.58 (584.94)	219.18 (395.19)	113.4 (− 78.55 to 305.35)
Specialist doctor	Surgical oncologist	60.13 (152.89)	80.53 (188.85)	− 20.4 (− 91.01 to 50.21)
	**Radiation oncologist**	**696.46 (1115.82)**	**1322.96 (1819.04)**	**− 626.5 (− 1233.3 to − 19.70)**
	Medical oncologist	130.32 (173.60)	268.17 (499.5)	− 137.84 (− 290.29 to 14.60)
	Other cancer specialists	51.03 (179.72)	30.95 (82.39)	20.07 (− 34.38 to 74.52)
	**Sub-total specialist doctor**	**937.95 (1258.38)**	**1702.62 (2072.99)**	**− 764.67 (− 1460.21 to − 69.13)**
Palliative care	Palliative care professional (doctor, nurse)	2.44 (17.23)	5.34 (25.89)	− 2.90 (− 11.68 to 5.89)
Allied health	Dietician	16.83 (69.60)	13.89 (40.98)	2.94 (− 19.05 to 24.93)
	Social worker	1.10 (7.79)	7.77 (46.38)	− 6.67 (− 21.21 to 7.88)
	Physiotherapist	201.5 (370.04)	130.33 (193.14)	71.18 (− 40.74 to 183.09)
	Psychologist	55.95 (164.13)	12.00 (36.03)	43.95 (− 3.16 to 91.07)
	Lymphoedema therapist	357.03 (560.71)	382.05 (814.29)	− 25.02 (− 298.20 to 248.16)
	Sub-total allied health services	632.42 (849.39)	546.03 (864.19)	86.39 (− 255.98 to 428.75)
Other surgery	Surgery (e.g. for local recurrence)	71.38 (181.53)	224.12 (751.88)	− 152.74 (− 373.35 to 67.88)
	Stereotactic radiosurgery (SRS)	0 (0)	103.74 (534.79)	na
	Sub-total other surgery	71.38 (181.53)	327.86 (910.12)	− 256.48 (− 513.61 to 0.66)
Diagnostic tests or scans	Cytology testing (e.g. biopsy)	48.03 (91.66)	43.21 (93.51)	4.82 (− 32.76 to 42.40)
	MRI scan	431.22 (697.81)	343 (558.14)	88.21 (− 157.10 to 333.53)
	CT scan	606.52 (908.13)	879.55 (1116.55)	− 273.03 (− 683.84 to 137.78)
	PET scan	148.29 (173.49)	192.58 (201.58)	− 44.29 (− 115.99 to 27.41)
	Blood test	1317.42 (1917.13)	1457.77 (1503.58)	− 140.35 (− 834.06 to 553.37)
	Ultrasound	259.51 (517.84)	159.45 (281.3)	100.07 (− 65.72 to 265.85)
	X-Ray	11.45 (45.25)	18.94 (70.76)	− 7.49 (− 31.46 to 16.48)
	Bone scan	12.11 (73.81)	20.48 (99.24)	− 8.36 (− 45.31 to 28.59)
	Mutation testing	46.57 (92.68)	28.05 (68.63)	18.52 (− 11.22 to 48.26)
	Other diagnostic tests	14.81 (66.06)	79.41 (343.87)	− 64.6 (− 165.01 to 35.82)
	Sub-total diagnostic tests or scans	2895.93 (3721.08)	3222.43 (2955.76)	− 326.49 (− 1666.83 to 1013.85)
Medicine	Medicine	573.24 (1678.55)	2552.97 (7482.93)	− 1979.73 (− 4165.2 to 205.74)
Radiation therapy and Systemic therapies	Radiation therapy	425.37 (1316.59)	447.12 (1202.62)	− 21.75 (− 527.32 to 483.82)
	Targeted therapy	4439.54 (23402.92)	4473.89 (23344.64)	− 34.35 (− 9300.67 to 9231.98)
	Chemotherapy	0 (0)	144.67 (508.31)	–
	Immunotherapy	1752.47 (4614.33)	2313.4 (7840.72)	− 560.93 (− 3091.79 to 1969.92)
	Sub-total therapies	6617.38 (25177.22)	7379.08 (24300.44)	− 761.70 (− 10608.16 to 9084.77)
Hospitalizations	Hospitalization costs	13,921.87 (26955.29)	16,434.07 (32963.43)	− 2512.26 (− 14,654.23 to 9629.7)
Total healthcare costs	**Healthcare costs**	26,555.02 (42498.35)	33,493.11 (45884.66)	− 6938.09 (− 24360.26 to 10484.09)

*SD* Standard deviation; *GP* General practitioner; *PET* Positron emission tomography

^¥^Randomized patient required only one-time index surgery (IL or I-IL) within 120 days

^§^Bootstrap confidence intervals with 1000 replications. A 5% discounted rate was applied in the cost estimation. Bold mark shows the significant difference

### Cost-utility Analysis

Table 2 shows the outcomes and costs by category and group allocation. In the base case analysis, per-patient healthcare costs of IL surgery were $6938 lower than for I-IL surgery ($26,555 vs. $ 33,493, 95% CI −$24,360 to $10,484). The IL procedure was associated with a nonsignificant increase in mean LYs (0.05, 95% CI −0.78 to 0.87) and mean QALYs (0.04, 95% CI –0.49 to 0.57) compared with the I-IL procedure (Table 2). This indicates the IL procedure was slightly more effective and less costly than the I-IL procedure (Table 2). When both incremental costs and LYs or QALYs were considered, the IL procedure was a dominant strategy for treating melanoma patients. The net monetary benefit for the IL procedure was $9438 per patient per LY saved, and $8938 per patient per QALY gained for the overall trial population, at a willingness to pay threshold of AU$50,000 per LY/QALY gained.

### Sensitivity Analysis

Deterministic sensitivity analyses to assess the robustness of results are presented in Supplementary Appendix Table S1.4. The sensitivity analysis results were consistent with our base-case analysis. The average cost per patient treated was consistently lower in the IL-surgery group than the I-IL group across all scenarios. The estimated ICER/ICURs based on these sensitivity analyses were dominant for LY saved and QALYs gained for IL compared with the I-IL surgery (Supplementary Appendix Table S1.4).

## Discussion

This is the first study to use randomized trial evidence that compares the costs and health outcomes of inguinal surgery approaches for stage III metastatic melanoma.^33^ Our study showed the average cost per patient treated was consistently lower in the IL surgery group than the I-IL surgery group. Health outcomes were also slightly but not significantly higher in the IL surgery group, representing a potential clinically meaningful improvement.^32^ The estimated ICUR/ICER showed IL surgery was dominant (i.e., more effective and less expensive) over the I-IL surgical procedure. Extensive sensitivity analyses supported these results.

A subgroup analysis of patients with inguinal disease randomized to surgery ± adjuvant radiation therapy, reported acceptable QoL, despite initial improvement and significant incidence of ongoing regional symptoms following IL or I-IL surgical procedures.^34^ Additional clinical data (e.g., deep lymph nodes) from an unpublished EAGLE FM substudy, which has been submitted for journal publication, revealed that among patients with no evidence of pelvic disease on PET/CT who underwent I-IL, six patients were found to have positive nodes on dissection. Concurrently, another EAGLE FM publication (Lee et al., 2024) in *Annals of Surgical Oncology* reported that the prevalence of leg lymphedema remained comparably high in both the IL and I-IL groups, with the highest rates observed at 6 months postprocedure.^35^ This study also highlighted that there were no significant baseline differences in pathology findings between the groups, nor were there significant associations with the total number of nodes removed, the presence of groin radiotherapy, wound infection rates, or the usage of compression garments.^35^ Importantly, this trial also reported no significant difference in overall survival, based on whether an IL or I-IL dissection was undertaken.^34^

Our study has notable strengths. First, use of a randomized comparison and preplanned best-practice methods for within-trial economic evaluation with 36 months of follow-up. Second, the study population was geographically diverse across six countries, increasing the generalizability of our findings. Third, the analytical exploration used assessments of costs related to healthcare use taken from participant-level data adjudicated by trial coordinators centrally. The study produced reliable findings based on unbiased healthcare use data from study records rather than self-reported data.^36^

Nevertheless, this study had some limitations. Although the point estimate of decreased costs and increased QALYs is clear, the statistical significance of both costs and QALYs at 36 months is uncertain, most likely owing to our small sample of 98 patients. However, the results from extensive sensitivity analyses are consistent with our base-case scenario. Findings of the economic evaluation from an Australian health system perspective may not be representative of other international health systems, although the engagement of international participants in this trial indicated that a similar treatment protocol was acceptable across different health system settings. This study was conducted from a health system perspective, focusing on health-related outcomes and direct medical costs. We excluded cost components, such as patient out-of-pocket expenses (e.g., transportation costs) or indirect costs (e.g., income loss due to work absenteeism and waiting time for medical appointments), borne by patients and their families. The inclusion of these components could present an opportunity to achieve a comprehensive understanding of broader societal costs.

### Implications of Findings

Our study underscores the importance of personalized and evidence-based treatment approaches, especially for patients with limited access to essential cancer medicines; those who do not respond to immunotherapy; and in settings where cutting-edge first-line treatments are either unavailable or prohibitively expensive.^37–39^ The findings suggest that in the current landscape of rapidly advancing adjuvant therapies, transitioning toward more effective or cost-efficient treatments, such as simpler surgical interventions, could lead to better patient outcomes. These approaches can support the efficient allocation of healthcare resources, supporting the overarching goals of enhancing care quality and efficiency within healthcare systems.

Given that effective systemic therapies in the adjuvant and neoadjuvant settings are now more widely available, we propose a de-implementation of I-IL surgery may be warranted. Due consideration should be given to whether an individual patient should be offered IL or I-IL surgery, knowing that the less involved IL has equivalent or potentially better outcomes in relation to surgical morbidity and cost-effectiveness. This shift in practice is supported by the increase of effective systemic therapies used in the adjuvant and more recently neoadjuvant setting,^3,7,9,40^ which may allow for faster transition to systemic treatments, reducing the need for more extensive surgery. Extensive surgery is likely to be used more for salvage after systemic therapy failure and these results are not directly relatable to that emerging scenario.

Data from this study suggest there is unlikely to be a health economic benefit from I-IL and therefore do not support the use of I-IL for Stage III metastatic melanoma to groin lymph nodes when PET/CT imaging shows no evidence of pelvic disease.

## Conclusions

Our study suggests that IL might be the preferred surgical strategy for treating stage III melanoma patients with groin metastases when the pretreatment PET/CT scan shows no evidence of pelvic node involvement. For any healthcare systems where systemic therapies are not available, IL may slightly improve quality-adjusted survival and reduce health system costs.

## Supplementary Information

Below is the link to the electronic supplementary material.

Supplementary file1 (DOCX 197 KB)

Supplementary file2 (DOCX 38 KB)
