# Correction: Economic Evaluation of Inguinal Versus Ilio-inguinal Lymphadenectomy for Patients with Stage III Metastatic Melanoma to Groin Lymph Nodes: Evidence from the EAGLE FM Randomized Trial

**DOI:** 10.1245/s10434-025-17337-2

**Published:** 2025-04-16

**Authors:** Rashidul Alam Mahumud, Chi Kin Law, Daniel Ariza Ospino, Johannes H. W. de Wilt, Barbara L. van Leeuwen, Chris Allan, Vinicius de Lima Vazquez, Rowan Pritchard Jones, Julie Howle, Barbara Peric, Andrew J. Spillane, Rachael Lisa Morton

**Affiliations:** 1 NHMRC Clinical Trials Centre, Faculty of Medicine and Health, The University of Sydney, Camperdown, NSW Australia; 2 Melanoma and Skin Cancer Trials Limited, Melbourne, VIC Australia; 3 Department of Surgery, Radboud University Medical Center, Nijmegen, The Netherlands; 4 Division of Surgical Oncology, Department of Surgery, University Medical Center Groningen, University of Groningen, Groningen, The Netherlands; 5 Faculty of Medicine, Mater Clinical School, University of Queensland, Brisbane, QLD Australia; 6 Molecular Oncology Research Center, Barretos Cancer Hospital, Barretos, São Paulo, Brazil; 7 Department of Surgery of Melanoma and Sarcoma, Barretos Cancer Hospital, São Paulo, Brazil; 8 Department of Molecular and Clinical Cancer Medicine, Institute of Systems, Molecular and Integrative Biology, University of Liverpool, Liverpool, UK; 9 Mersey and West Lancashire Teaching Hospitals NHS Trust, Prescot, Knowsley, UK; 10 Sydney Medical School, The University of Sydney, Camperdown, NSW Australia; 11 Institute of Oncology Ljubljana, Zaloska 2, 1000 Ljubljana, Slovenia; 12 Faculty of Medicine, University of Ljubljana, Vrazov trg 2, 1000 Ljubljana, Slovenia; 13 Translational Research Hub, Poche Centre, Melanoma Institute Australia, The University of Sydney, Wollstonecraft, NSW Australia; 14 Breast and Melanoma Surgery Unit, Royal North Shore Hospital, St Leonards, NSW Australia; 15 Mater Hospital, Wollstonecraft, NSW Australia

**Correction to: Annals of Surgical Oncology** 10.1245/s10434-025-17040-2

In the original online version of this article, Barbara Peric was inadvertently omitted from the author group. The original article was corrected.

