# Supplementary material for: Economic Evaluation of Inguinal Versus Ilio-inguinal Lymphadenectomy for Patients with Stage III Metastatic Melanoma to Groin Lymph Nodes: Evidence from the EAGLE FM Randomized Trial
Source: Ann Surg Oncol. 2025 Feb 27;32(6):4211–22. doi: 10.1245/s10434-025-17040-2 (PMC12049375; doi:10.1245/s10434-025-17040-2)
Supplement: Supplementary file 1 — Supplementary file1 (DOCX 197 KB) [file 10434_2025_17040_MOESM1_ESM.docx]

Supplementary Appendix S1

This appendix formed part of the original submission.

Economic evaluation of inguinal versus ilio-inguinal lymphadenectomy for patients with Stage III metastatic melanoma to groin lymph nodes: evidence from the EAGLE FM randomized trial

**Authors:** Rashidul Alam Mahumud, PhD, MCncrSc, MPH, MSc^1^; Chi Kin Law, PhD, BFin^1^; Daniel Ariza Ospino, MPH ^2^; Johannes H.W. de Wilt, MD, PhD^3^; Barbara L. van Leeuwen, MD, PhD^;^, Chris Allan, MBBS, MPhil, FRACS^5^; Vinicius de Lima Vazquez, MD, PhD^6,7^; Rowan Pritchard Jones, FRCS(Plast) MD(Bris), MAMAA^8,9^; Julie Howle, MD^10^; Andrew J Spillane, MD, FRACS^10,11,12,13^; Rachael Lisa Morton, PhD, MScMed(Clin Epi)(Hons)^1,11^

**Affiliations:**

^1^NHMRC Clinical Trials Centre, Faculty of Medicine and Health, The University of Sydney, Camperdown, NSW, 2006, Australia.

^2^Melanoma and Skin Cancer Trials Limited, Melbourne, VIC, 3004, Australia.

^3^Department of Surgery, Radboud University Medical Center, Nijmegen, the Netherlands.

^4^Division of Surgical Oncology, Department of Surgery, University Medical Center Groningen, University of Groningen, Hanzeplein 1, 9713 GZ Groningen, The Netherlands.

^5^Faculty of Medicine, Mater Clinical School, University of Queensland, Brisbane, QLD-4072, Australia.

^6^Molecular Oncology Research Center, Barretos Cancer Hospital, Antenor Duarte Villela, 1331, Barretos, São Paulo Zip Code: 14784 400, Brazil

^7^Department of Surgery of Melanoma and Sarcoma, Barretos Cancer Hospital, São Paulo, Brazil.

^8^Department of Molecular and Clinical Cancer Medicine, Institute of Systems, Molecular and Integrative Biology, University of Liverpool, UK

^9^Mersey and West Lancashire Teaching Hospitals NHS Trust, Prescot, Knowsley, UK;

^10^Sydney Medical School, The University of Sydney, Camperdown, NSW, 2006, Australia

^11^Melanoma Institute Australia, Poche Centre, The University of Sydney, Translational Research Hub, Wollstonecraft, NSW, 2065, Australia.

^12^Breast and Melanoma Surgery Unit, Royal North Shore Hospital, St Leonards, NSW, 2065, Australia

^13^Mater Hospital, Wollstonecraft, NSW 2060

# Supplementary Table S1.1 CHEERS 2022 Checklist

| **Topic** | **No.** | **Item** | **Location where item is reported** |
| --- | --- | --- | --- |
| **Title** |  |  |  |
|  | 1 | Identify the study as an economic evaluation and specify the interventions being compared. | Please see title page |
| **Abstract** |  |  |  |
|  | 2 | Provide a structured summary that highlights context, key methods, results, and alternative analyses. | We provided a structured summary on page 3, which captured required contexts. |
| **Introduction** |  |  |  |
| **Background and objectives** | 3 | Give the context for the study, the study question, and its practical relevance for decision making in policy or practice. | We included an introduction section and outlined the objectives and rationale for this economic evaluation. Please refer to pages 4 and 5. |
| **Methods** |  |  |  |
| **Health economic analysis plan** | 4 | Indicate whether a health economic analysis plan was developed and where available. | We have incorporated health economics components to generate economic evidence for policymakers based on funding requirements. The proposed analysis plan for the health economic components has been revised in accordance with the advice of the trial management committee. However, we have not yet created a health economic analysis plan. Please refer to the trial registry for more details. Trial registration: NCT02166788; ACTRN12614000721606 |
| **Study population** | 5 | Describe characteristics of the study population (such as age range, demographics, socioeconomic, or clinical characteristics). | Please see pages 5-6 and Table 1 (Describe characteristics of the study population). |
| **Setting and location** | 6 | Provide relevant contextual information that may influence findings. | please see page 6 |
| **Comparators** | 7 | Describe the interventions or strategies being compared and why chosen. | please see page 6. |
| **Perspective** | 8 | State the perspective(s) adopted by the study and why chosen. | Please see page 7. |
| **Time horizon** | 9 | State the time horizon for the study and why appropriate. | please see page 8. |
| **Discount rate** | 10 | Report the discount rate(s) and reason chosen. | A 5% discount rate for outcomes was applied after one year according to Australian guidelines. please see pages 8 and 9. |
| **Selection of outcomes** | 11 | Describe what outcomes were used as the measure(s) of benefit(s) and harm(s). | Outcome selection, measurement, and valuation were reported in pages 7 and 8. |
| **Measurement of outcomes** | 12 | Describe how outcomes used to capture benefit(s) and harm(s) were measured. | please see pages 7 and 8. |
| **Valuation of outcomes** | 13 | Describe the population and methods used to measure and value outcomes. | please see pages 7 and 8. |
| **Measurement and valuation of resources and costs** | 14 | Describe how costs were valued. | please see page 9. |
| **Currency, price date, and conversion** | 15 | Report the dates of the estimated resource quantities and unit costs, plus the currency and year of conversion. | please see page 9. |
| **Rationale and description of model** | 16 | If modelling is used, describe in detail and why used. Report if the model is publicly available and where it can be accessed. | N/A |
| **Analytics and assumptions** | 17 | Describe any methods for analysing or statistically transforming data, any extrapolation methods, and approaches for validating any model used. | please see page 10. |
| **Characterising heterogeneity** | 18 | Describe any methods used for estimating how the results of the study vary for subgroups. | please see page 10. |
| **Characterising distributional effects** | 19 | Describe how impacts are distributed across different individuals or adjustments made to reflect priority populations. | please see page 10. |
| **Characterising uncertainty** | 20 | Describe methods to characterise any sources of uncertainty in the analysis. | please see page 10. |
| **Approach to engagement with patients and others affected by the study** | 21 | Describe any approaches to engage patients or service recipients, the general public, communities, or stakeholders (such as clinicians or payers) in the design of the study. | please see pages 9 and 10. |
| **Results** |  |  |  |
| **Study parameters** | 22 | Report all analytic inputs (such as values, ranges, references) including uncertainty or distributional assumptions. | please see pages 11-12. |
| **Summary of main results** | 23 | Report the mean values for the main categories of costs and outcomes of interest and summarise them in the most appropriate overall measure. | please see page 13. |
| **Effect of uncertainty** | 24 | Describe how uncertainty about analytic judgments, inputs, or projections affect findings. Report the effect of choice of discount rate and time horizon, if applicable. | please see page 14. |
| **Effect of engagement with patients and others affected by the study** | 25 | Report on any difference patient/service recipient, general public, community, or stakeholder involvement made to the approach or findings of the study | please see pages 13-15. |
| **Discussion** |  |  |  |
| **Study findings, limitations, generalisability, and current knowledge** | 26 | Report key findings, limitations, ethical or equity considerations not captured, and how these could affect patients, policy, or practice. | please see pages 14-16. |
| **Other relevant information** |  |  |  |
| **Source of funding** | 27 | Describe how the study was funded and any role of the funder in the identification, design, conduct, and reporting of the analysis | please see page 17 |
| **Conflicts of interest** | 28 | Report authors conflicts of interest according to journal or International Committee of Medical Journal Editors requirements. | please see page 17. |

*From:* Husereau D, Drummond M, Augustovski F, et al. Consolidated Health Economic Evaluation Reporting Standards 2022 (CHEERS 2022) Explanation and Elaboration: A Report of the ISPOR CHEERS II Good Practices Task Force. Value Health 2022;25. <doi:10.1016/j.jval.2021.10.008>

**Supplementary Table S1.2 Undiscounted estimate of quality-adjusted survival by surgery group**

| Follow-up times after surgery (T) | IL | | | | | I-IL | | | | |
| --- | --- | --- | --- | --- | --- | --- | --- | --- | --- | --- |
|  | Survival probability (S_t_) | Life year (LY)^1^ | Health utility values (Q_t_) | Average health utility values (AQ_t_) | Quality-adjusted survival (QAS) | Survival probability (S_t_) | Life year (LY)^1^ | Health utility values (Q_t_) | Average health utility values (AQ_t_) | Quality-adjusted survival (QAS) |
| (1) | (2) | (3) | (4) | (5) | (6) = (3) × (5) | (7) | (8) | (9) | (10) | (11) = (8) × (10) |
| Baseline (0) | 1.00 | - | 0.79186 |  |  | 1.00 | - | 0.784208 | - | - |
| 3 months (0.25 years) | 1.00 | 0.25 | 0.74128 | 0.76657 | 0.1916425 | 1.00 | 0.25 | 0.698313 | 0.7412604 | 0.1853151 |
| 6 months (0.5 years) | 1.00 | 0.25 | 0.75112 | 0.7462 | 0.18655 | 0.9792 | 0.2474 | 0.739674 | 0.7189932 | 0.1778789 |
| 9 months (0.75 years) | 0.96 | 0.245 | 0.718957 | 0.7350387 | 0.1800845 | 0.9375 | 0.2395875 | 0.727233 | 0.7334533 | 0.1757262 |
| 12 months (1 year) | 0.92 | 0.235 | 0.73314 | 0.7260485 | 0.1706214 | 0.9167 | 0.231775 | 0.767139 | 0.7471861 | 0.173179 |
| 15 months (1.25 years) | 0.88 | 0.225 | 0.753976 | 0.7435579 | 0.1673005 | 0.9167 | 0.229175 | 0.795634 | 0.7813868 | 0.1790743 |
| 18 months (1.5 years) | 0.859 | 0.27375 | 0.775725 | 0.7648506 | 0.1662594 | 0.875 | 0.2239625 | 0.772128 | 0.7838812 | 0.17556 |
| 21 months (1.75 years) | 0.859 | 0.21475 | 0.717368 | 0.7465467 | 0.1603209 | 0.8333 | 0.2135375 | 0.790744 | 0.7814359 | 0.1668659 |
| 24 months (2 years) | 0.8364 | 0.211925 | 0.818861 | 0.7681148 | 0.1627827 | 0.8333 | 0.208325 | 0.778744 | 0.7847436 | 0.1634817 |
| 30 months (2.5 years) | 0.8364 | 0.4182 | 0.760629 | 0.7897449 | 0.3302713 | 0.7895 | 0.4057 | 0.753806 | 0.7662746 | 0.3108776 |
| 36 months (3 years) | 0.8138 | 0.41255 | 0.816588 | 0.7886084 | 0.3253404 | 0.745 | 0.383625 | 0.775118 | 0.7644616 | 0.2932666 |
| Total QAS per patient | | | | | 2.041174 |  | | | | 2.001225 |

LY = [(S_t_+S_t+1_)/2) × (T_t+1_ – T_t_)], AQ_t_ = (Q_t_ + Q_t+1_)/2, The Australian EQ-5D-5L weights were used to measure utility values at these time points (Norman et al. 2013).

**Supplementary Table S1.3 Healthcare resource use**

| **Category of healthcare services** | **Items** | **Medicare Benefits Schedule (MBS) (2021 reference year)** |
| --- | --- | --- |
| **Index surgery** | Index surgery | 36502, 30330 [inguinal lymphadenectomy(30330), ilio-inguinal lymphadenectomy (30330 + 36502)]. |
| **General practitioner** | General practitioner clinic visit | 53 |
|  | General practitioner home visit | 5003 |
|  | General practitioner (telephone) | 5003, 5000 |
|  | Other health professional visits | 104, 105, 10958, 10970, 82200, 10970, |
| Specialist doctor | Radiation oncologist | 104, 105 |
|  | Medical oncologist | 116, 132, 133 |
|  | Surgical oncologist | 104, 133, |
|  | Other cancer specialist visits | 82200, 10970, 39720 |
| Palliative care | Palliative care professional (i.e., doctor, nurse) | 3005 |
| Allied health | Dietician | 10954 |
|  | Social worker | 80150 |
|  | Physiotherapist | 10960 |
|  | Psychologist | 80150 |
|  | Lymphoedema therapist | 10960, 10560 |
| Other surgery | Surgery (e.g. for local recurrence) | 31376, 31373 |
|  | Stereotactic radiosurgery (SRS) | 15600 |
| Diagnostic imaging and tests | Cytology testing (e.g., biopsy) | 73049, 73059, 30071, 73049, |
|  | MRI scan | 56807, 63454 |
|  | CT scan | 1115, 56807, 56047, 56407, 56507, 56412, 56307, 56219, 56107, 56401, 56301, 56625 |
|  | PET scan | 61553, 66500, 65070 |
|  | Blood test | 65070, 66500, 65070, 66500, |
|  | Ultrasound | 55816, 55832, 55824, 55011, 55014, 55816, 55732, 55832, 55014, |
|  | X-Ray | 57509, 57512, 57515, 57518, 57521, 57524, 57527, 57506 |
|  | Bone scan | 61425 |
|  | Mutation Testing | 73336 |
|  | Other diagnostic tests | e.g., 30071 |
| Radiotherapy | Radiotherapy | R64Z |
| Systemic therapies | Targeted therapy | 61553 |
|  | Chemotherapy | 34540 |
|  | Immunotherapy | 11626X, 10436G (PBS item). |
| [2021 Pharmaceutical Benefits Scheme (PBS)](https://www.pbs.gov.au/pbs/home) **[(2021 reference year)](https://www.pbs.gov.au/pbs/home)** | | |
| 11186R, 2418G, 2417F, 2751T, 2752W, 12002Q, 3300Q, 1886G, 12002Q, 1888J, 11933C, 3300Q, 11998L, 11933C, 3300Q, 4076M, 1081X, 5008N, 5006L, 3390K, 9440W, 4131K, 8604W, 8605X, 11845K, 1153Q, 3318P, 12115P, 5477G, 1799Q, 11227X, 10778G, 4175R, 1585K, 1209P, 8220P, 8738X, 8318T, 5057E, 12054K, 2507Y, 5356X, 1300K, 12010D, 8480H, 1272Y, 1357K, 3322W, 10102Q, 1370D, 8263X, 8510X, 8701Y, 8600P, 2488Y, 4237B, 10605E, 1527J, 10579T, 12222G, 2412Y, 2824P, 9302N, 1499X, 1500Y. 5118J, 3192B, 10250L, 11241H, 1558B, 8359Y, 11576G, 3095X, 3318P, 1590Q, 8654L, 2174K, 11366F, 8213G, 11135C, 2732T, 8203R, 5146W, 3118D, 2430X, 8055Y, 12507G, 1324Q, 1642K, 1621H, 9029F, 8513C, 12549L, 1795L, 8171C, 8600P, 1594X, 8410P, 5471Y, 12048D, 5194J, 12074L, 9400R, 12210P, 3486L, 3051N, 8704D, 9006B, 9007C, 8695P, 1480X, 1936X, 1948M, 2565B, 1955X, 1978D, 5260W, 12305P, 9243L, 1501B, 2339D, 2951H, 10102Q, 5221T, 8526R, 1418P, 11280Q, 12024W, 11364D, 2269K, 3114X, 3113W, 2844Q, 2211J, 10516L, 5535H, 8213G, 8214H, 8521L, 8606Y, 8297Q, 8296P, 11112W, 8854B, 10169F, 4004R, 11820D, 11291G, 10061M, 2507Y, 11303X, 10579T, 4592Q, 1466E, 8401E, 3192B, 4134N, 8248D, 2174K, 9287T, 2318B, 11739W, 8733P, 8043H, 8627C, 9019Q, 8298R, 5573H, 13817F, 8008L, 8399C, 3050M, 1916W, 2355Y, 2335X, 2348N, 2268J, 2691P, 2606E, 2574L, 2628H, 8837D, 3391L, 4070F, 8356T, 3484J, 12024W, 10382K, 8597L, 9371F, 4043T, 1358L, 4200C, 5520M, 8465M, 2951H, 1527J, 2175L, 11933C, 4175R, 1119X, 9008D, 3116M, 9332E, 10797G, 2174K, 10156M, 2971J, 2565B, 8763F, 11933C, 5552F, 12041R, 10127B, 2708M, 10605E, 2622B, 1324Q, 3393N, 11205R, 1081X. | | |
| Immunotherapy: 11626X, 10436G | | |

*GP = General practitioner, PET = positron emission tomography*

**Supplementary Table S1.4 Sensitivity analysis testing robustness of results**

| Parameter, scenario, and cost-utility analysis | Surgery Group | | Difference |  |
| --- | --- | --- | --- | --- |
|  | **Inguinal lymphadenectomy (IL)** | **Ilio-inguinal lymphadenectomy (I-IL)** | **Mean (95% CI)** |  |
| Base case | | | | |
| Undiscounted | | | | |
| Total direct medical cost, mean (SD) (AU$) | 27187 (44403) | 34190 (46909) | -7004 (-24978 to 10971)^§^ |  |
| Mean life years (LYs) (SD) | 2.68 (0.23) | 2.63 (0.28) | 0.05 (-0.53 to 0.63) |  |
| QALY, mean (using Norman et al. 2013 Australian utility values) | 2.04 | 2.00 | 0.04 (-0.32 to 0.40) |  |
| QALY, mean (using Norman et al. 2022 Australian utility values) | 2.37 | 2.35 | 0.02 (-0.45 to 0.49) |  |
| Cost-effectiveness for LY gained | IL dominates | | |  |
| Cost-effectiveness (using Norman et al. 2013 Australian utility values) | IL dominates | | |  |
| Cost-effectiveness (using Norman et al. 2022 Australian utility values) | IL dominates | | |  |
| Incremental Net Benefit | | | | |
| - At a WTP threshold of AU$ 50000 per life year gained | 9504 | | |  |
| - At a WTP threshold of AU$ 50000 per QALY gained (using Norman et al. 2013 Australian utility values) | 9504 | | |  |
| - At a WTP threshold of AU$ 50000 per QALY gained (using Norman et al. 2022 Australian utility values) | 8004 | | |  |
| Discount rate at 5% | | | | |
| (1) 10% increase in general practitioner costs (+10%) | | | | |
| Total direct medical cost, mean (SD) (AU$) | 26588 (42527) | 33515 (45885) | -6927 (-24353 to 10499)^§^ |  |
|  |  |  |  |  |
| QALY, mean (using Norman et al. 2013 Australian utility values) | 1.95 | 1.91 | 0.04 (-0.49 to 0.57) |  |
| QALY, mean (using Norman et al. 2022 Australian utility values) | 2.27 | 2.25 | 0.02 (-0.68 to 0.72) |  |
| Cost-effectiveness for LY gained | IL dominates | | |  |
| Cost-effectiveness (using Norman et al. 2013 Australian utility values) | IL dominates | | |  |
| Cost-effectiveness (using Norman et al. 2022 Australian utility values) | IL dominates | | |  |
| Incremental Net Benefit, mean (AU$) | | | | |
| - At a WTP threshold of AU$ 50000 per life year gained | 9427 | | |  |
| - At a WTP threshold of AU$ 50000 per QALY gained (using Norman et al. 2013 Australian utility values) | 8927 | | |  |
| - At a WTP threshold of AU$ 50000 per QALY gained (using Norman et al. 2022 Australian utility values) | 7927 | | |  |
| (2) 10% decrease in general practitioner costs (-10%) | | | | |
| Total direct medical cost, mean (SD) (AU$) | 26522 (42470) | 33471 (45885) | -6949 (-24368 to 10469)^§^ |  |
| Cost-effectiveness for LY gained | IL dominates | | |  |
| Cost-effectiveness (using Norman et al. 2013 Australian utility values) | IL dominates | | |  |
| Cost-effectiveness (using Norman et al. 2022 Australian utility values) | IL dominates | | |  |
| Incremental Net Benefit, mean (AU$) | | | | |
| - At a WTP threshold of AU$ 50000 per life year gained | 9449 | | |  |
| - At a WTP threshold of AU$ 50000 per QALY gained (using Norman et al. 2013 Australian utility values) | 8949 | | |  |
| - At a WTP threshold of AU$ 50000 per QALY gained (using Norman et al. 2022 Australian utility values) | 7949 | | |  |
| (3) 20% increase in general practitioner costs (+20%) | | | | |
| Total direct medical cost, mean (SD) (AU$) | 26622 (42555) | 33537 (45884) | -6915 (-24346 to 10515)^§^ |  |
| Cost-effectiveness for LY gained | IL dominates | | |  |
| Cost-effectiveness (using Norman et al. 2013 Australian utility values) | IL dominates | | |  |
| Cost-effectiveness (using Norman et al. 2022 Australian utility values) | IL dominates | | |  |
| Incremental Net Benefit, mean (AU$) | | | | |
| - At a WTP threshold of AU$ 50000 per life year gained | 9415 | | |  |
| - At a WTP threshold of AU$ 50000 per QALY gained (using Norman et al. 2013 Australian utility values) | 8915 | | |  |
| - At a WTP threshold of AU$ 50000 per QALY gained (using Norman et al. 2022 Australian utility values) | 7915 | | |  |
| (4) 20% decrease in general practitioner costs (-20%) | | | | |
| Total direct medical cost, mean (SD) (AU$) | 26489 (42442) | 33449 (45885) | -6753 (-24375 to 10453)^§^ |  |
| Cost-effectiveness for LY gained | IL dominates | | |  |
| Cost-effectiveness (using Norman et al. 2013 Australian utility values) | IL dominates | | |  |
| Cost-effectiveness (using Norman et al. 2022 Australian utility values) | IL dominates | | |  |
| Incremental Net Benefit, mean (AU$) | | | | |
| - At a WTP threshold of AU$ 50000 per life year gained | 9253 | | |  |
| - At a WTP threshold of AU$ 50000 per QALY gained (using Norman et al. 2013 Australian utility values) | 8960 | | |  |
| - At a WTP threshold of AU$ 50000 per QALY gained (using Norman et al. 2022 Australian utility values) | 7960 | | |  |
| (5) 30% increase in general practitioner costs (+30%) | | | | |
| Total direct medical cost, mean (SD) (AU$) | 26655 (42584) | 33559 (45884) | -6696 (-24338 to 10530)^§^ |  |
| Cost-effectiveness for LY gained | IL dominates | | |  |
| Cost-effectiveness (using Norman et al. 2013 Australian utility values) | IL dominates | | |  |
| Cost-effectiveness (using Norman et al. 2022 Australian utility values) | IL dominates | | |  |
| Incremental Net Benefit, mean (AU$) | | | | |
| - At a WTP threshold of AU$ 50000 per life year gained | 9296 | | |  |
| - At a WTP threshold of AU$ 50000 per QALY gained (using Norman et al. 2013 Australian utility values) | 8904 | | |  |
| - At a WTP threshold of AU$ 50000 per QALY gained (using Norman et al. 2022 Australian utility values) | 7904 | | |  |
| (6) 30% decrease in general practitioner costs (-30%) | | | | |
| Total direct medical cost, mean (SD) (AU$) | 26455 (42414) | 33427 (45885) | -6972 (-24382 to 10438)^§^ |  |
| Cost-effectiveness for LY gained | IL dominates | | |  |
| Cost-effectiveness (using Norman et al. 2013 Australian utility values) | IL dominates | | |  |
| Cost-effectiveness (using Norman et al. 2022 Australian utility values) | IL dominates | | |  |
| Incremental Net Benefit, mean (AU$) | | | | |
| - At a WTP threshold of AU$ 50000 per life year gained | 9472 | | |  |
| - At a WTP threshold of AU$ 50000 per QALY gained (using Norman et al. 2013 Australian utility values) | 8972 | | |  |
| - At a WTP threshold of AU$ 50000 per QALY gained (using Norman et al. 2022 Australian utility values) | 7972 | | |  |

Note: WTP = willingness-to-pay, QALY= quality-adjusted survival, SD = standard deviation, ^§^CI = bootstrap 95% confidence interval with 1000 replications

**Supplementary Table S1.4 Sensitivity analysis testing robustness of results** (Continued)

| Parameter, scenario, and cost-utility analysis | Surgery Group | | Difference |  |
| --- | --- | --- | --- | --- |
|  | **Inguinal lymphadenectomy (IL)** | **Ilio-inguinal lymphadenectomy (I-IL)** | **Mean (95% CI)** |  |
| (1) 10% increase in specialist doctor costs (+10%) | | | | |
| Total direct medical cost, mean (SD) (AU$) | 26649 (42574) | 33663 (45939) | -7015 (-24464 to 10435)^§^ |  |
| Cost-effectiveness for LY gained | IL dominates | | |  |
| Cost-effectiveness (using Norman et al. 2013 Australian utility values) | IL dominates | | |  |
| Cost-effectiveness (using Norman et al. 2022 Australian utility values) | IL dominates | | |  |
| Incremental Net Benefit, mean (AU$) | | | | |
| - At a WTP threshold of AU$ 50000 per life year gained | 9515 | | |  |
| - At a WTP threshold of AU$ 50000 per QALY gained (using Norman et al. 2013 Australian utility values) | 9014 | | |  |
| - At a WTP threshold of AU$ 50000 per QALY gained (using Norman et al. 2022 Australian utility values) | 8015 | | |  |
| (2) 10% decrease in specialist doctor costs (-10%) | | | | |
| Total direct medical cost, mean (SD) (AU$) | 25856 (41967) | 31839 (45340) | -5984 (-23158 to 11191)^§^ |  |
| Cost-effectiveness for LY gained | IL dominates | | |  |
| Cost-effectiveness (using Norman et al. 2013 Australian utility values) | IL dominates | | |  |
| Cost-effectiveness (using Norman et al. 2022 Australian utility values) | IL dominates | | |  |
| Incremental Net Benefit, mean (AU$) | | | | |
| - At a WTP threshold of AU$ 50000 per life year gained | 8484 | | |  |
| - At a WTP threshold of AU$ 50000 per QALY gained (using Norman et al. 2013 Australian utility values) | 7984 | | |  |
| - At a WTP threshold of AU$ 50000 per QALY gained (using Norman et al. 2022 Australian utility values) | 6984 | | |  |
| (3) 20% increase in specialist doctor costs (+20%) | | | | |
| Total direct medical cost, mean (SD) (AU$) | 26743 (42650) | 33834 (45995) | -7091 (-24569 to 10387)^§^ |  |
| Cost-effectiveness for LY gained | IL dominates | | |  |
| Cost-effectiveness (using Norman et al. 2013 Australian utility values) | IL dominates | | |  |
| Cost-effectiveness (using Norman et al. 2022 Australian utility values) | IL dominates | | |  |
| Incremental Net Benefit, mean (AU$) | | | | |
| - At a WTP threshold of AU$ 50000 per life year gained | 9591 | | |  |
| - At a WTP threshold of AU$ 50000 per QALY gained (using Norman et al. 2013 Australian utility values) | 9091 | | |  |
| - At a WTP threshold of AU$ 50000 per QALY gained (using Norman et al. 2022 Australian utility values) | 8091 | | |  |
| (4) 20% decrease of specialist doctor costs (-20%) | | | | |
| Total direct medical cost, mean (SD) (AU$) | 25762 (41895) | 31669 (45296) | -5907 (-23057 to 11243)^§^ |  |
| Cost-effectiveness for LY gained | IL dominates | | |  |
| Cost-effectiveness (using Norman et al. 2013 Australian utility values) | IL dominates | | |  |
| Cost-effectiveness (using Norman et al. 2022 Australian utility values) | IL dominates | | |  |
| Incremental Net Benefit, mean (AU$) | | | | |
| - At a WTP threshold of AU$ 50000 per life year gained | 8407 | | |  |
| - At a WTP threshold of AU$ 50000 per QALY gained (using Norman et al. 2013 Australian utility values) | 7907 | | |  |
| - At a WTP threshold of AU$ 50000 per QALY gained (using Norman et al. 2022 Australian utility values) | 6907 | | |  |
| (5) 30% increase in specialist doctor costs (+30%) | | | | |
| Total direct medical cost, mean (SD) (AU$) | 26836 (42727) | 34004 (46051) | -7167 (-24673 to 10338)^§^ |  |
| Cost-effectiveness for LY gained | IL dominates | | |  |
| Cost-effectiveness (using Norman et al. 2013 Australian utility values) | IL dominates | | |  |
| Cost-effectiveness (using Norman et al. 2022 Australian utility values) | IL dominates | | |  |
| Incremental Net Benefit, mean (AU$) | | | | |
| - At a WTP threshold of AU$ 50000 per life year gained | 9667 | | |  |
| - At a WTP threshold of AU$ 50000 per QALY gained (using Norman et al. 2013 Australian utility values) | 9167 | | |  |
| - At a WTP threshold of AU$ 50000 per QALY gained (using Norman et al. 2022 Australian utility values) | 8167 | | |  |
| (6) 30% decrease in specialist doctor costs (-30%) | | | | |
| Total direct medical cost, mean (SD) (AU$) | 25668 (41822) | 31499 (45253) | -5831 (-22956 to 11295)^§^ |  |
| Cost-effectiveness for LY gained | IL dominates | | |  |
| Cost-effectiveness (using Norman et al. 2013 Australian utility values) | IL dominates | | |  |
| Cost-effectiveness (using Norman et al. 2022 Australian utility values) | IL dominates | | |  |
| Incremental Net Benefit, mean (AU$) | | | | |
| - At a WTP threshold of AU$ 50000 per life year gained | 8331 | | |  |
| - At a WTP threshold of AU$ 50000 per QALY gained (using Norman et al. 2013 Australian utility values) | 7831 | | |  |
| - At a WTP threshold of AU$ 50000 per QALY gained (using Norman et al. 2022 Australian utility values) | 6831 | | |  |

Note: WTP = willingness-to-pay, QALY= quality-adjusted survival, SD = standard deviation, ^§^CI = bootstrap 95% confidence interval with 1000 replications

**Supplementary Table S1.4 Sensitivity analysis testing robustness of results** (Continued)

| Parameter, scenario, and cost-utility analysis | Surgery Group | | Difference |  |
| --- | --- | --- | --- | --- |
|  | **Inguinal lymphadenectomy (IL)** | **Ilio-inguinal lymphadenectomy (I-IL)** | **Mean (95% CI)** |  |
| (1) 10% increase in diagnostic tests or scans costs (+10%) | | | | |
| Total direct medical cost, mean (SD) (AU$) | 26845 (42714) | 33815 (46086) | -6971 (-24477 to 10535)^§^ |  |
| Cost-effectiveness for LY gained | IL dominates | | |  |
| Cost-effectiveness (using Norman et al. 2013 Australian utility values) | IL dominates | | |  |
| Cost-effectiveness (using Norman et al. 2022 Australian utility values) | IL dominates | | |  |
| Incremental Net Benefit, mean (AU$) | | | | |
| - At a WTP threshold of AU$ 50000 per life year gained | 9471 | | |  |
| - At a WTP threshold of AU$ 50000 per QALY gained (using Norman et al. 2013 Australian utility values) | 8971 | | |  |
| - At a WTP threshold of AU$ 50000 per QALY gained (using Norman et al. 2022 Australian utility values) | 7971 | | |  |
| (2) 10% decrease in diagnostic tests or scans costs (-10%) | | | | |
| Total direct medical cost, mean (SD) (AU$) | 26265 (42285) | 33171 (45685) | -6905 (-24245 to 10434)^§^ |  |
| Cost-effectiveness for LY gained | IL dominates | | |  |
| Cost-effectiveness (using Norman et al. 2013 Australian utility values) | IL dominates | | |  |
| Cost-effectiveness (using Norman et al. 2022 Australian utility values) | IL dominates | | |  |
| Incremental Net Benefit, mean (AU$) | | | | |
| - At a WTP threshold of AU$ 50000 per life year gained | 9405 | | |  |
| - At a WTP threshold of AU$ 50000 per QALY gained (using Norman et al. 2013 Australian utility values) | 8905 | | |  |
| - At a WTP threshold of AU$ 50000 per QALY gained (using Norman et al. 2022 Australian utility values) | 7905 | | |  |
| (3) 20% increase in diagnostic tests or scans costs (+20%) | | | | |
| Total direct medical cost, mean (SD) (AU$) | 27134 (42932) | 34138 (46288) | -7003 (-24594 to 10587)^§^ |  |
| Cost-effectiveness for LY gained | IL dominates | | |  |
| Cost-effectiveness (using Norman et al. 2013 Australian utility values) | IL dominates | | |  |
| Cost-effectiveness (using Norman et al. 2022 Australian utility values) | IL dominates | | |  |
| Incremental Net Benefit, mean (AU$) | | | | |
| - At a WTP threshold of AU$ 50000 per life year gained | 9503 | | |  |
| - At a WTP threshold of AU$ 50000 per QALY gained (using Norman et al. 2013 Australian utility values) | 9003 | | |  |
| - At a WTP threshold of AU$ 50000 per QALY gained (using Norman et al. 2022 Australian utility values) | 8003 | | |  |
| (4) 20% decrease in diagnostic tests or scans costs (-20%) | | | | |
| Total direct medical cost, mean (SD) (AU$) | 25976 (42073) | 32849 (45486) | -6873 (-24130 to 10384)^§^ |  |
| Cost-effectiveness for LY gained | IL dominates | | |  |
| Cost-effectiveness (using Norman et al. 2013 Australian utility values) | IL dominates | | |  |
| Cost-effectiveness (using Norman et al. 2022 Australian utility values) | IL dominates | | |  |
| Incremental Net Benefit, mean (AU$) | | | | |
| - At a WTP threshold of AU$ 50000 per life year gained | 9373 | | |  |
| - At a WTP threshold of AU$ 50000 per QALY gained (using Norman et al. 2013 Australian utility values) | 8873 | | |  |
| - At a WTP threshold of AU$ 50000 per QALY gained (using Norman et al. 2022 Australian utility values) | 7873 | | |  |
| (5) 30% increase in diagnostic tests or scans costs (+30%) | | | | |
| Total direct medical cost, mean (SD) (AU$) | 27424 (43152) | 34460 (46491) | -7036 (-24711 to 10639)^§^ |  |
| Cost-effectiveness for LY gained | IL dominates | | |  |
| Cost-effectiveness (using Norman et al. 2013 Australian utility values) | IL dominates | | |  |
| Cost-effectiveness (using Norman et al. 2022 Australian utility values) | IL dominates | | |  |
| Incremental Net Benefit, mean (AU$) | | | | |
| - At a WTP threshold of AU$ 50000 per life year gained | 9536 | | |  |
| - At a WTP threshold of AU$ 50000 per QALY gained (using Norman et al. 2013 Australian utility values) | 9036 | | |  |
| - At a WTP threshold of AU$ 50000 per QALY gained (using Norman et al. 2022 Australian utility values) | 8036 | | |  |
| (6) 30% decrease in diagnostic tests or scans costs (-30%) | | | | |
| Total direct medical cost, mean (SD) (AU$) | 25686 (41864) | 32526 (45288) | -6840 (-24015 to 10335)^§^ |  |
| Cost-effectiveness for LY gained | IL dominates | | |  |
| Cost-effectiveness (using Norman et al. 2013 Australian utility values) | IL dominates | | |  |
| Cost-effectiveness (using Norman et al. 2022 Australian utility values) | IL dominates | | |  |
| Incremental Net Benefit, mean (AU$) | | | | |
| - At a WTP threshold of AU$ 50000 per life year gained | 9340 | | |  |
| - At a WTP threshold of AU$ 50000 per QALY gained (using Norman et al. 2013 Australian utility values) | 8840 | | |  |
| - At a WTP threshold of AU$ 50000 per QALY gained (using Norman et al. 2022 Australian utility values) | 7840 | | |  |

Note: WTP = willingness-to-pay, QALY= quality-adjusted survival, SD = standard deviation, ^§^CI = bootstrap 95% confidence interval with 1000 replications

**Supplementary Table S1.4 Sensitivity analysis testing robustness of results** (Continued)

| **Parameter, scenario, and cost-utility analysis** | **Surgery Group** | | **Difference** |
| --- | --- | --- | --- |
|  | **Inguinal lymphadenectomy (IL)** | **Ilio-inguinal lymphadenectomy (I-IL)** | **Mean (95% CI)** |
| (1) 10% increase in Systemic and radiotherapy costs (+10%) | | | |
| Total direct medical cost, mean (SD) (AU$) | 27217 (44342) | 34231 (47284) | -7014 (-25057 to 11029)^§^ |
| Cost-effectiveness for LY gained | IL dominates | | |
| Cost-effectiveness (using Norman et al. 2013 Australian utility values) | IL dominates | | |
| Cost-effectiveness (using Norman et al. 2022 Australian utility values) | IL dominates | | |
| Incremental Net Benefit, mean (AU$) | | | |
| - At a WTP threshold of AU$ 50000 per life year gained | 9514 | | |
| - At a WTP threshold of AU$ 50000 per QALY gained (using Norman et al. 2013 Australian utility values) | 9014 | | |
| - At a WTP threshold of AU$ 50000 per QALY gained (using Norman et al. 2022 Australian utility values) | 8014 | | |
| (2) 10% decrease in Systemic and radiotherapy costs (-10%) | | | |
| Total direct medical cost, mean (SD) (AU$) | 25893 (40727) | 32755 (44574) | -6862 (-23698 to 9974)^§^ |
| Cost-effectiveness for LY gained | IL dominates | | |
| Cost-effectiveness (using Norman et al. 2013 Australian utility values) | IL dominates | | |
| Cost-effectiveness (using Norman et al. 2022 Australian utility values) | IL dominates | | |
| Incremental Net Benefit, mean (AU$) | | | |
| - At a WTP threshold of AU$ 50000 per life year gained | 9362 | | |
| - At a WTP threshold of AU$ 50000 per QALY gained (using Norman et al. 2013 Australian utility values) | 8862 | | |
| - At a WTP threshold of AU$ 50000 per QALY gained (using Norman et al. 2022 Australian utility values) | 7862 | | |
| (3) 20% increase in Systemic and radiotherapy costs (+20%) | | | |
| Total direct medical cost, mean (SD) (AU$) | 27879 (46249) | 34969 (48764) | -7090 (-25786 to 11605)^§^ |
| Cost-effectiveness for LY gained | IL dominates | | |
| Cost-effectiveness (using Norman et al. 2013 Australian utility values) | IL dominates | | |
| Cost-effectiveness (using Norman et al. 2022 Australian utility values) | IL dominates | | |
| Incremental Net Benefit, mean (AU$) | | | |
| - At a WTP threshold of AU$ 50000 per **life year** gained | 9590 | | |
| - At a WTP threshold of AU$ 50000 per QALY gained (using Norman et al. 2013 Australian utility values) | 9090 | | |
| - At a WTP threshold of AU$ 50000 per QALY gained (using Norman et al. 2022 Australian utility values) | 8090 | | |
| (4) 20% decrease in Systemic and radiotherapy costs (-20%) | | | |
| Total direct medical cost, mean (SD) (AU$) | 25232 (39038) | 32017 (43361) | -6786 (-23074 to 9502)^§^ |
| Cost-effectiveness for LY gained | IL dominates | | |
| Cost-effectiveness (using Norman et al. 2013 Australian utility values) | IL dominates | | |
| Cost-effectiveness (using Norman et al. 2022 Australian utility values) | IL dominates | | |
| Incremental Net Benefit, mean (AU$) | | | |
| - At a WTP threshold of AU$ 50000 per life year gained | 9286 | | |
| - At a WTP threshold of AU$ 50000 per QALY gained (using Norman et al. 2013 Australian utility values) | 8786 | | |
| - At a WTP threshold of AU$ 50000 per QALY gained (using Norman et al. 2022 Australian utility values) | 7786 | | |
| (5) 30% increase in Systemic and radiotherapy costs (+30%) | | | |
| Total direct medical cost, mean (SD) (AU$) | 28540 (48213) | 35707 (50318) | -7167 (-26542 to 12209)^§^ |
| Cost-effectiveness for LY gained | IL dominates | | |
| Cost-effectiveness (using Norman et al. 2013 Australian utility values) | IL dominates | | |
| Cost-effectiveness (using Norman et al. 2022 Australian utility values) | IL dominates | | |
| Incremental Net Benefit, mean (AU$) | | | |
| - At a WTP threshold of AU$ 50000 per life year gained | 9667 | | |
| - At a WTP threshold of AU$ 50000 per QALY gained (using Norman et al. 2013 Australian utility values) | 9167 | | |
| - At a WTP threshold of AU$ 50000 per QALY gained (using Norman et al. 2022 Australian utility values) | 8167 | | |
| (6) 30% decrease in Systemic and radiotherapy costs (-30%) | | | |
| Total direct medical cost, mean (SD) (AU$) | 24570 (37442) | 31279 (42252) | -6710 (-22492 to 9072)^§^ |
| Cost-effectiveness for LY gained | IL dominates | | |
| Cost-effectiveness (using Norman et al. 2013 Australian utility values) | IL dominates | | |
| Cost-effectiveness (using Norman et al. 2022 Australian utility values) | IL dominates | | |
| Incremental Net Benefit, mean (AU$) | | | |
| - At a WTP threshold of AU$ 50000 per life year gained | 9210 | | |
| - At a WTP threshold of AU$ 50000 per QALY gained (using Norman et al. 2013 Australian utility values) | 8710 | | |
| - At a WTP threshold of AU$ 50000 per QALY gained (using Norman et al. 2022 Australian utility values) | 7710 | | |

Note: WTP = willingness-to-pay, QALY= quality-adjusted survival, SD = standard deviation, ^§^CI = bootstrap 95% confidence interval with 1000 replications

**Supplementary Table S1.4 Sensitivity analysis testing robustness of results** (Continued)

| **Parameter, scenario, and cost-utility analysis** | **Surgery Group** | | **Difference** |  |
| --- | --- | --- | --- | --- |
|  | **Inguinal lymphadenectomy (IL)** | **Ilio-inguinal lymphadenectomy (I-IL)** | **Mean (95% CI)** |  |
| (1) 10% increase in hospitalisation costs (+10%) | | | | |
| Total direct medical cost, mean (SD) (AU$) | 27947 (44601) | 35137 (48631) | -7189 (-25586 to 11207)^§^ |  |
| Cost-effectiveness for LY gained | IL dominates | | |  |
| Cost-effectiveness (using Norman et al. 2013 Australian utility values) | IL dominates | | |  |
| Cost-effectiveness (using Norman et al. 2022 Australian utility values) | IL dominates | | |  |
| Incremental Net Benefit, mean (AU$) | | | | |
| - At a WTP threshold of AU$ 50000 per life year gained | 9689 | | |  |
| - At a WTP threshold of AU$ 50000 per QALY gained (using Norman et al. 2013 Australian utility values) | 9190 | | |  |
| - At a WTP threshold of AU$ 50000 per QALY gained (using Norman et al. 2022 Australian utility values) | 8190 | | |  |
| (2) 10% decrease in hospitalisation costs (-10%) | | | | |
| Total direct medical cost, mean (SD) (AU$) | 25163 (40466) | 31850 (43216) | -6687 (-23166 to 9793)^§^ |  |
| Cost-effectiveness for LY gained | IL dominates | | |  |
| Cost-effectiveness (using Norman et al. 2013 Australian utility values) | IL dominates | | |  |
| Cost-effectiveness (using Norman et al. 2022 Australian utility values) | IL dominates | | |  |
| Incremental Net Benefit, mean (AU$) | | | | |
| - At a WTP threshold of AU$ 50000 per life year gained | 9187 | | |  |
| - At a WTP threshold of AU$ 50000 per QALY gained (using Norman et al. 2013 Australian utility values) | 8687 | | |  |
| - At a WTP threshold of AU$ 50000 per QALY gained (using Norman et al. 2022 Australian utility values) | 7687 | | |  |
| (3) 20% increase in hospitalisation costs (+20%) | | | | |
| Total direct medical cost, mean (SD) (AU$) | 29339 (46765) | 36780 (51441) | -7441 (-26839 to 11958)^§^ |  |
| Cost-effectiveness for LY gained | IL dominates | | |  |
| Cost-effectiveness (using Norman et al. 2013 Australian utility values) | IL dominates | | |  |
| Cost-effectiveness (using Norman et al. 2022 Australian utility values) | IL dominates | | |  |
| Incremental Net Benefit, mean (AU$) | | | | |
| - At a WTP threshold of AU$ 50000 per life year gained | 9941 | | |  |
| - At a WTP threshold of AU$ 50000 per QALY gained (using Norman et al. 2013 Australian utility values) | 9441 | | |  |
| - At a WTP threshold of AU$ 50000 per QALY gained (using Norman et al. 2022 Australian utility values) | 8441 | | |  |
| (4) 20% decrease in hospitalisation costs (-20%) | | | | |
| Total direct medical cost, mean (SD) (AU$) | 23771 (38515) | 30206 (40639) | -6436 (-22010 to 9139)^§^ |  |
| Cost-effectiveness for LY gained | IL dominates | | |  |
| Cost-effectiveness (using Norman et al. 2013 Australian utility values) | IL dominates | | |  |
| Cost-effectiveness (using Norman et al. 2022 Australian utility values) | IL dominates | | |  |
| Incremental Net Benefit, mean (AU$) | | | | |
| - At a WTP threshold of AU$ 50000 per life year gained | 8936 | | |  |
| - At a WTP threshold of AU$ 50000 per QALY gained (using Norman et al. 2013 Australian utility values) | 8436 | | |  |
| - At a WTP threshold of AU$ 50000 per QALY gained (using Norman et al. 2022 Australian utility values) | 7436 | | |  |
| (5) 30% increase in hospitalisation costs (+30%) | | | | |
| Total direct medical cost, mean (SD) (AU$) | 30732 (48981) | 38423 (54307) | -7692 (-28115 to 12731)^§^ |  |
| Cost-effectiveness for LY gained | IL dominates | | |  |
| Cost-effectiveness (using Norman et al. 2013 Australian utility values) | IL dominates | | |  |
| Cost-effectiveness (using Norman et al. 2022 Australian utility values) | IL dominates | | |  |
| Incremental Net Benefit, mean (AU$) | | | | |
| - At a WTP threshold of AU$ 50000 per life year gained | 10192 | | |  |
| - At a WTP threshold of AU$ 50000 per QALY gained (using Norman et al. 2013 Australian utility values) | 9692 | | |  |
| - At a WTP threshold of AU$ 50000 per QALY gained (using Norman et al. 2022 Australian utility values) | 8692 | | |  |
| (6) 30% decrease in hospitalisation costs (-30%) | | | | |
| Total direct medical cost, mean (SD) (AU$) | 22378 (36658) | 28563 (38173) | -6184 (-20898 to 8530)^§^ |  |
| Cost-effectiveness for LY gained | IL dominates | | |  |
| Cost-effectiveness (using Norman et al. 2013 Australian utility values) | IL dominates | | |  |
| Cost-effectiveness (using Norman et al. 2022 Australian utility values) | IL dominates | | |  |
| Incremental Net Benefit, mean (AU$) | | | | |
| - At a WTP threshold of AU$ 50000 per life year gained | 8684 | | |  |
| - At a WTP threshold of AU$ 50000 per QALY gained (using Norman et al. 2013 Australian utility values) | 8184 | | |  |
| - At a WTP threshold of AU$ 50000 per QALY gained (using Norman et al. 2022 Australian utility values) | 7184 | | |  |

Note: WTP = willingness-to-pay, QALY= quality-adjusted survival, SD = standard deviation, ^§^CI = bootstrap 95% confidence interval with 1000 replications

**Supplementary Table S1.4** Sensitivity analysis testing robustness of results (Continued)

| Parameter, scenario, and cost-utility analysis | Surgery Group | | Difference |
| --- | --- | --- | --- |
|  | **Inguinal lymphadenectomy (IL)** | **Ilio-inguinal lymphadenectomy**  **(I-IL)** | **Mean (95% CI)** |
| Discount rate at 3% | | | |
| Total direct medical cost, mean (SD) (AU$) | 26798 (43222) | 33762 (46277) | -6964 (-24596 to 10668)^§^ |
| QALY, mean (using Norman et al. 2013 Australian utility values) | 1.98 | 1.95 | 0.03 (-0.29 to 0.35) |
| QALY, mean (using Norman et al. 2022 Australian utility values) | 2.31 | 2.29 | 0.02 (-0.41 to 0.45) |
| Cost-effectiveness for LY gained | IL dominates | | |
| Cost-effectiveness (using Norman et al. 2013 Australian utility values) | IL dominates | | |
| Cost-effectiveness (using Norman et al. 2022 Australian utility values) | IL dominates | | |
| Incremental Net Benefit, mean (AU$) | | | |
| - At a WTP threshold of AU$ 50000 per QALY gained (using Norman et al. 2013 Australian utility values) | 8464 | | |
| - At a WTP threshold of AU$ 50000 per QALY gained (using Norman et al. 2022 Australian utility values) | 7964 | | |
| Discount rate at 3.5% | | | |
| Total direct medical cost, mean (SD) (AU$) | 26736 (43036) | 33693 (46177) | -6958 (-24536 to 10621)^§^ |
| QALY, mean (using Norman et al. 2013 Australian utility values) | 1.98 | 1.94 | 0.04 (-0.29 to 0.37) |
| QALY, mean (using Norman et al. 2022 Australian utility values) | 2.30 | 2.28 | 0.02 (-0.43 to 0.47) |
| Cost-effectiveness for LY gained |  | | |
| Cost-effectiveness (using Norman et al. 2013 Australian utility values) | IL dominates | | |
| Cost-effectiveness (using Norman et al. 2022 Australian utility values) | IL dominates | | |
| Incremental Net Benefit, mean (AU$) | | | |
| - At a WTP threshold of AU$ 50000 per QALY gained (using Norman et al. 2013 Australian utility values) | 8958 | | |
| - At a WTP threshold of AU$ 50000 per QALY gained (using Norman et al. 2022 Australian utility values) | 7958 | | |

Note: WTP = willingness-to-pay, QALY = quality-adjusted life year, SD = standard deviation, ^§^CI = bootstrap 95% confidence interval with 1000 replications

Supplementary Appendix Figure S1 Completed EQ-5D-5L data with no missing values across all five dimensions
