# Supplementary material for: Economic Evaluation of Inguinal Versus Ilio-inguinal Lymphadenectomy for Patients with Stage III Metastatic Melanoma to Groin Lymph Nodes: Evidence from the EAGLE FM Randomized Trial
Source: Ann Surg Oncol. 2025 Feb 27;32(6):4211–22. doi: 10.1245/s10434-025-17040-2 (PMC12049375; doi:10.1245/s10434-025-17040-2)
Supplement: Supplementary file 2 — Supplementary file2 (DOCX 38 KB) [file 10434_2025_17040_MOESM2_ESM.docx]

**Supplementary Appendix file S2:**

Supplement to Mahumud et al. Economic evaluation of inguinal versus ilio-inguinal lymphadenectomy for patients with Stage III metastatic melanoma to groin lymph nodes: evidence from the EAGLE FM randomized trial

**Calculation Procedure for Health Utility Scores Based on the EQ-5D-5L Instrument Among the EAGLE FM Trial Population**

**An instrument used to collect quality of life data: EQ-5D-5L** (EuroQol 5-Dimension, 5-Level):

The **EQ-5D-5L** instrument is a widely used, standardised instrument designed to measure health-related quality of life (HRQoL). It is used in clinical studies, health economics evaluations, and population health surveys. This instrument provides a simple and generic measure of health that is applicable across a diverse range of diseases, conditions, and demographic groups.

**Calculation of Health Utility Scores:** We used the following procedure to compute health utility scores within the EAGLE FM trial population:

**Step 1:** Understanding the health states and levels within the EQ-5D-5L

The descriptive system of the EQ-5D-5L captures health status across five dimensions, each describing a key aspect of daily life:

1. Dimension-1 (Mobility): Ability to move around.
2. Dimension-2 (Self-Care): Ability to wash, dress, and take care of oneself.
3. Dimension-3 (Usual Activities): Ability to perform work, study, housework, leisure activities, etc.
4. Dimension-4 (Pain/Discomfort): Experience of physical pain or discomfort.
5. Dimension-5 (Anxiety/Depression): Experience of anxiety or depression.

Each dimension features **five** severity levels:

- Level 1 (No problems)
- Level 2 (Slight problems)
- Level 3 (Moderate problems)
- Level 4 (Severe problems)
- Level 5 (Extreme problems)

If a patient selects one level per dimension, which produces a unique health state. For example:

- Health state **11111** represents no problems in any dimension (perfect health).
- Health state **12345** indicates slight problems with self-care, moderate problems with usual activities, severe pain/discomfort, and extreme anxiety/depression.

**Step 2: Health utility scoring of EQ-5D-5L**

- Health state encoding:

Each health state is encoded as a 5-digit number corresponding to the selected level for each dimension (e.g., 12345).

- Utility Values (Population Tariffs):

Utility scores were derived from the Australian population **tariff** (**value set)** based on population preferences.^1^ In general, the tariff assigns a single utility value to each health state across the EQ-5D-5L instrument, with **1.0** = perfect health (**11111**), zero (0) = State equivalent to death and value less than 0 indicating states considered worse than death.

Utility values were computed by applying disutility decrements related to the severity levels within each dimension, accompanied by an additional decrement (N_5_) for extremely severe health states. If a patient reports an extreme health problem during a specific follow-up period, an additional decrement (N_5_) is incorporated into the disutility calculation. Furthermore, if multiple extreme health problems are reported within the same follow-up period, the additional decrement (N_5_) is adjusted only once for that period.

- Example calculation of utility scores:

Consider a patient completes the EQ-5D-5L questionnaire at a 12-month follow-up:

- Mobility: Level 2 (slight problems)
- Self-care: Level 1 (no problems)
- Usual Activities: Level 3 (moderate problems)
- Pain/Discomfort: Level 4 (severe problems)
- Anxiety/Depression: Level 5 (extreme problems)

This response pattern corresponds to health state 21345. Using the Australian tariff^1^,

- Calculate disutilities for the reported dimensions: 0.072+0.000+0.120+0.259+0.398 = 0.849

We must adjust an additional decrement, whereas this patient reported extreme anxiety or depression (level 5).

- Adjust with an additional decrement for total disutility = 0.849 + 0.059 = 0.908
- The utility score was then calculated as:

Utility score = **1.0 -** (total disutilities for dimensions with adjusted additional decrements).

= 1.0 – 0.908 = 0.092

This methodological procedure was consistently applied across all subsequent follow-up periods within the trial population.

**Step 3:** Calculation of mean utility scores (using a complete case) by trial allocation groups [inguinal lymphadenectomy, IL and ilio-inguinal lymphadenectomy, I-IL] and follow-up periods.

| Mean utility scores (Qi) (complete case) ^¥^ by trial allocation groups [IL and I-IL] | | | | |
| --- | --- | --- | --- | --- |
| Trial follow-up period (months) | IL surgery group | | I-IL surgery group | |
|  | Mean | Sd | Mean | Sd |
| Baseline | 0.792583 | 0.20867 | 0.78424 | 0.22297 |
| 3 | 0.741326 | 0.23212 | 0.69836 | 0.19694 |
| 6 | 0.75114 | 0.22844 | 0.73966 | 0.19143 |
| 9 | 0.718951 | 0.31936 | 0.72727 | 0.23854 |
| 12 | 0.73315 | 0.37589 | 0.76715 | 0.1684 |
| 15 | 0.75397 | 0.26699 | 0.79559 | 0.1714 |
| 18 | 0.775686 | 0.24788 | 0.77214 | 0.19968 |
| 21 | 0.717412 | 0.30449 | 0.7907 | 0.19267 |
| 24 | 0.818849 | 0.20629 | 0.77871 | 0.22279 |
| 30 | 0.760618 | 0.2904 | 0.7538 | 0.25513 |
| 36 | 0.816533 | 0.30561 | 0.77513 | 0.26198 |

^¥^in this analysis, only patient who completed the EQ-5D-5L quality of life instrument without any missing values for the specified follow-up period were included. .

**Step 4:** Dealing missing values in the calculation of mean utility scores (Qi) by trial allocation groups [IL and I-IL] and consecutive follow-up periods.

We used a single imputation method to handle missing data, applying a single predicted value for estimating health utilities, such as the mean, by trial allocation group with corresponding follow-up periods for a given case.^2^

For instance, Mr X (patient) completed the EQ-5D-5L questionnaire at baseline, 3-month, 6-month, 9-month, and 15-month follow-up periods. However, due to missed clinical visits or severe health conditions, as noted in the trial records, Mr X could not provide quality of life (QoL) data at the 12-month follow-up, which we then considered as a missing case at 12 months period. In this scenario, we calculated the mean utility scores for complete cases (please see step 3, described above) by trial allocation groups and follow-up points. Subsequently, we imputed the mean utility score for Mr. X at 12-month follow-up. This approach was similarly applied to other patients with missing cases where applicable.

| Mean utility scores (Qi) (with imputation) by trial allocation groups [IL and I-IL] | | | | |
| --- | --- | --- | --- | --- |
| Trial follow-up period (months) | IL surgery group | | I-IL surgery group | |
|  | Mean (Qi) | Sd | Mean (Qi) | Sd |
| Baseline | 0.79186 | 0.204291 | 0.784208 | 0.208249 |
| 3 | 0.74128 | 0.214904 | 0.698313 | 0.183941 |
| 6 | 0.75112 | 0.21149 | 0.739674 | 0.187126 |
| 9 | 0.718957 | 0.297801 | 0.727233 | 0.220847 |
| 12 | 0.73314 | 0.362218 | 0.76714 | 0.160185 |
| 15 | 0.753976 | 0.235871 | 0.795634 | 0.162604 |
| 18 | 0.775725 | 0.231444 | 0.772128 | 0.188879 |
| 21 | 0.717368 | 0.287559 | 0.790744 | 0.176803 |
| 24 | 0.818861 | 0.197255 | 0.778744 | 0.210738 |
| 30 | 0.760629 | 0.286099 | 0.753806 | 0.25146 |
| 36 | 0.816588 | 0.286488 | 0.775118 | 0.245587 |

These estimated mean utility scores were used to calculate the undiscounted quality-adjusted survival (QAS) for each trial allocation group across various follow-up times post-surgery (please see equation 3 for details)

References:

1 Norman R, Cronin P, Viney R. A pilot discrete choice experiment to explore preferences for EQ-5D-5L health states. *Appl Health Econ Health Policy* 2013; **11**: 287–298.

2 Gabrio A, Mason AJ, Baio G. Handling missing data in within-trial cost-effectiveness analysis: A review with future recommendations. *PharmacoEconomics - Open* 2017; **1**: 79–97.
